# Parenting after a history of childhood maltreatment: A scoping review and map of evidence in the perinatal period

**DOI:** 10.1371/journal.pone.0213460

**Published:** 2019-03-13

**Authors:** Catherine Chamberlain, Graham Gee, Stephen Harfield, Sandra Campbell, Sue Brennan, Yvonne Clark, Fiona Mensah, Kerry Arabena, Helen Herrman, Stephanie Brown, for the ‘Healing the Past by Nurturing the Future’ group

**Affiliations:** 1 Judith Lumley Centre, La Trobe University, Melbourne, Victoria, Australia; 2 Murdoch Children’s Research Institute, Melbourne, Victoria, Australia; 3 School of Public Health and Preventive Medicine, Monash University, Melbourne, Victoria, Australia; 4 Victorian Aboriginal Health Service, Melbourne, Victoria, Australia; 5 Wardliparingga Aboriginal Research Unit, South Australian Health and Medical Research Institute, Adelaide, South Australia, Australia; 6 School of Public Health, The University of Adelaide, Adelaide, South Australia, Australia; 7 Sansom Institute for Health Research, The University of South Australia, Adelaide, South Australia, Australia; 8 Centre for Chronic Disease Prevention, James Cook University, Cairns, Queensland, Australia; 9 Centre for Indigenous Health Equity Research, Central Queensland University, Cairns, Queensland, Australia; 10 School of Public Health and Preventive Medicine, Monash University, Melbourne, Victoria, Australia; 11 School of Psychology, University of Adelaide, Hughes, Adelaide, South Australia, Australia; 12 South Australian Health and Medical Research Institute, Adelaide, South Australia, Australia; 13 Royal Children’s Hospital, Melbourne, Victoria, Australia; 14 Department of Paediatrics, University of Melbourne, Melbourne, Victoria, Australia; 15 Melbourne School of Population and Global Health, University of Melbourne, Melbourne, Victoria, Australia; 16 Centre for Youth Mental Health, The University of Melbourne, Melbourne, Victoria, Australia; 17 Orygen, The National Centre of Excellence in Youth Mental Health, Melbourne, Victoria, Australia; 18 Department of General Practice, University of Melbourne, Melbourne, Victoria, Australia; Università degli Studi di Perugia, ITALY

## Abstract

**Background and aims:**

Child maltreatment is a global health priority affecting up to half of all children worldwide, with profound and ongoing impacts on physical, social and emotional wellbeing. The perinatal period (pregnancy to two years postpartum) is critical for parents with a history of childhood maltreatment. Parents may experience ‘triggering’ of trauma responses during perinatal care or caring for their distressed infant. The long-lasting relational effects may impede the capacity of parents to nurture their children and lead to intergenerational cycles of trauma. Conversely, the perinatal period offers a unique life-course opportunity for parental healing and prevention of child maltreatment. This scoping review aims to *map perinatal evidence* regarding theories, intergenerational pathways, parents’ views, interventions and measurement tools involving parents with a history of maltreatment in their own childhoods.

**Methods and results:**

We searched Medline, Psychinfo, Cinahl and Embase to 30/11/2016. We screened 6701 articles and included 55 studies (74 articles) involving more than 20,000 parents. Most studies were conducted in the United States (42/55) and involved mothers only (43/55). *Theoretical constructs* include: attachment, social learning, relational-developmental systems, family-systems and anger theories; ‘hidden trauma’, resilience, post-traumatic growth; and ‘Child Sexual Assault Healing’ and socioecological models. Observational studies illustrate sociodemographic and mental health protective and risk factors that mediate/moderate *intergenerational pathways* to parental and child wellbeing. Qualitative studies provide rich descriptions of *parental experiences and views* about healing strategies and support. We found no specific *perinatal interventions* for parents with childhood maltreatment histories. However, several parenting interventions included elements which address parental history, and these reported positive effects on parent wellbeing. We found twenty-two *assessment tools* for identifying parental childhood maltreatment history or impact.

**Conclusions:**

Perinatal evidence is available to inform development of strategies to support parents with a history of child maltreatment. However, there is a paucity of applied evidence and evidence involving fathers and Indigenous parents.

## Introduction

Child maltreatment is a global health priority affecting 25 to 50% of children worldwide [1] and can have profound and ongoing impacts on physical and social and emotional wellbeing and development [2, 3]. Conflicting infant attachment and defence (fear or ‘flight, fright and freeze’) systems can be activated in response to child maltreatment which can lead to internal confusion and behavioural responses that are an attempt to manage distress and promote self-regulation, but may also result in increased confusion and harm [4]. These responses can be maintained into adulthood as part of a cluster of symptoms associated with recently proposed criteria for diagnosis of complex post-traumatic stress disorder (complex PTSD) [5]. Complex PTSD, caused by cumulative exposure to traumatic experiences that often involve interpersonal violation within a child’s care giving system (sometimes referred to as ‘developmental’ or ‘relational’ trauma), can occur in families, within the context of social institutions [6], and be exaberbated by cumulative traumatic experiences as an adult.

Long-term associations with childhood maltreatment include smoking, eating disorders, adolescent [7] and unplanned pregnancies [8], adverse birth outcomes [9], and a range of physical and psychological morbidities [10]. Critically, these long-lasting relational effects can impede the capacity of parents to nurture and care for children, leading to ‘intergenerational cycles’ of trauma [4]. Parental fear responses can be triggered by their child’s distress, and are often re-experienced as conflicting sensations and emotions, rather than as a thought-out narrative [11]. This in turn can give rise to hostile or helpless responses to the growing child’s needs [12]. In addition, the intimate nature of some perinatal experiences associated with pregnancy, birth and breastfeeding pose a high risk of triggering childhood trauma responses.

Conversely, the transition to parenthood during the perinatal period (pregnancy to two years postpartum) offers a unique life-course opportunity for improving health [13], for healing [3] and for relational growth [14]. Growing research into the ‘neurobiology of attachment’ demonstrates that healing can occur despite severe experiences of maltreatment, by restoring a sense of safety and well-being through nurturing, supportive relationships with others—a transition sometimes referred to as ‘earned security’. A positive strengths-based focus during the often-optimistic perinatal period has the potential to disrupt the ‘vicious cycle’ of intergenerational trauma into a ‘virtuous cycle’ that contains positively reinforcing elements that promote healing [14].

Despite the critical importance of the perinatal period for parents who have a history of maltreatment in their own childhoods; and frequent scheduled contacts with service providers during pregnancy, birth and early parenthood; there is limited specific guidance for care in the perinatal period for parents who have experienced maltreatment in their own childhoods [15]. In Australia, National Trauma Guidelines emphasize the need for trauma-informed care and trauma-specific support for the general population [6], and Perinatal Mental Health Guidelines recommend that perinatal care providers conduct a psychosocial assessment for mothers, including childhood maltreatment history [16]. However, there is limited guidance on trauma-specific care and support interventions in the perinatal period for parents with a history of childhood maltreatment, which is required for any population-based screening program [17].

Child maltreatment is not randomly distributed. The World Health Organization (WHO) use a socioecological framework [18] to explain why some people are at higher risk of experiencing interpersonal violence [19]. Parents with a history of childhood maltreatment are also more likely to have multiple socio-economic challenges, including unintended pregnancies [8], antenatal and postnatal depression [20], contact with the justice system and low employment [21]. Within Australia, there have been harrowing reports documenting high rates of child maltreatment and violence among Aboriginal and Torres Strait Islander (Aboriginal) communities in Australia [22]. The reasons for this are complex and lie beyond the scope of this review but are influenced by a legacy of past governmental policies of forced removal of Aboriginal children from their Aboriginal families and communities, intergenerational effects of these previous separations from family and culture, ongoing oppressive policies, social exclusion, marginalisation and poverty [23, 24]. Thus, Australian National Trauma Guidelines emphasize the need for understandings of complex trauma to be contextualised within socioecological environments [6].

The primary aim of this scoping review is to map perinatal evidence involving parents with a history of childhood maltreatment. Our specific purpose is to identify relevant evidence to support the co-design of strategies for perinatal trauma-informed care (awareness), recognition, assessment and support for Aboriginal parents with a history of maltreatment in their own childhoods. We briefly illustrate how this scoping review is being incorporated into the co-design process in the discussion, with a project led by our team [25]. While this review is designed to address a direct practical purpose, we expect that it will have broader applicability to those working with parents who have experienced maltreatment in their own childhoods and help to prioritise future research in this critical area. A secondary aim is to use this scoping review to refine the search strategy and develop detailed protocols for further in-depth systematic reviews (see S1 Appendix for overview of planned reviews).

The specific questions for this ‘phase 1’ scoping review are:

1. What theories are used during the perinatal period to understand and frame the impact of a parental history of childhood maltreatment?
2. What risk and protective factors are identified in epidemiological evidence of life-course and intergenerational pathways (mediators/moderators) between a parental history of childhood maltreatment and behavioural/health outcomes for parents and their infants?
3. What are the perinatal experiences of parents with a history of childhood maltreatment? And what perinatal strategies do these parents report using to heal and prevent intergenerational transmission of trauma to their child?
4. What perinatal interventions are described to support parents with a history of childhood maltreatment to improve parental and child wellbeing?
5. What tools have been reported in the perinatal period to identify parents with a history of childhood maltreatment and/or assess symptoms of complex trauma?

## Methods

PRISMA guidelines for systematic reviews informed the reporting of this scoping review (see S2 Appendix for checklist) and the protocol on which it is based (S3 Appendix). We have also referred to extension statements for scoping reviews [26] and equity-focussed reviews [27].

### Criteria for inclusion

**Participants:** Prospective (pre-pregnancy), pregnant and new parents (mothers and/or fathers) or families caring for children up to two years after birth. Where mean ages only were reported (and it was unclear if children were two years of age or younger), studies reporting a mean age of less than five years only (i.e. preschool age) were included. Studies which reported a proportion of participants as parents of children aged two years or less were also included, to err towards inclusivity. Despite the primary aim of the research being to inform co-design of strategies to support Aboriginal parents, we have not restricted the inclusion criteria to Aboriginal or Indigenous parents for several reasons. Firstly, we know there will be very limited published Indigenous-specific evidence available. Second, we will incorporate evidence from other population groups in a comprehensive co-design process which draws on the knowledge of Aboriginal parents and key-stakeholders, and enables Aboriginal Australian parents to consider the relevance of this evidence for them.

**Interventions/exposures:** Any parental report of childhood maltreatment. There is no broadly accepted consensus on a definition for complex PTSD, and this lack of consensus will be reflected in previously published included studies in this review. Therefore, for the purposes of this review, we are using the WHO definition of a key antecedent, child maltreatment:

“abuse and neglect that occurs to children under 18 years of age. It includes all types of physical and/or emotional ill-treatment, sexual abuse, neglect, negligence and commercial or other exploitation, which results in actual or potential harm to the child’s health, survival, development or dignity in the context of a relationship of responsibility, trust or power. Exposure to intimate partner violence is also sometimes included as a form of child maltreatment.”

[1]

We also include ‘proxy measures’ such as child protection substantiations or removal from their family of origin.

**Study type/comparisons:** Any study design, including randomised controlled studies (RCTs), cluster RCTs, cohort studies (including measurement/assessment studies), economic evaluations or qualitative studies (see S1 Appendix for an overview of the study types considered relevant to each question). We excluded reviews, guidelines, discussion and opinion papers, government reports and non-peer reviewed reports of primary studies. Review articles were screened for additional primary studies.

**Outcomes:** Theories; risk or protective factors which mediate or moderate parental or child outcomes; experiences, perspectives and strategies parents use for healing or preventing intergenerational transmission of trauma; acceptability, effectiveness and cost of current perinatal interventions; and perinatal tools used for assessing symptoms of complex trauma or exposure to childhood maltreatment. Studies which only reported associations between parental childhood maltreatment history and outcomes, without any investigation of mediating/moderating factors, were not included in this review as these associations have been well-established in other reviews [28].

### Search methods

We searched the following databases: Psychinfo, Medline, Cinahl and Embase up to 30/11/2016. The search terms included both thesaurus (MeSH) and keyword synonyms for ‘child abuse’ AND ‘intergenerational’ AND ‘prevention’ AND ‘parent’, using a search strategy developed and piloted in Psychinfo (see S4 Appendix) and subsequently modified for use in the remaining databases. We checked reference lists from relevant reviews identified from the search for additional potentially relevant primary studies.

References were downloaded into bibliographic reference management software (Endnote) and de-duplicated. One reviewer (CC) independently screened titles and abstracts to identify potentially relevant studies using an over-inclusive approach. The full texts of all potentially relevant studies were independently assessed by two reviewers (CC/GG) for inclusion, based on the pre-specified criteria for inclusion. Discrepancies were resolved by discussion.

### Data extraction

A data extraction tool was developed and piloted in Microsoft Excel by two reviewers (CC/SB). Two reviewers (CC & GG/SG/YV/SC/SB) independently extracted data on the items that follow.

**Population:** study setting; selection criteria; recruitment process and sample size; parenting stage (pre-pregnancy, pregnancy to six weeks postpartum, six weeks to one year postpartum, one to two years postpartum); type of childhood trauma reported; parent characteristics that relate to progress-plus ‘equity’ criteria relevant to this review population (‘at risk’ status, age, place of residence, race/ethnicity, gender, language, religion, socio-economic status, social capital (e.g. marital status), and other (e.g. mental illness)) [29].

**Intervention/study detail coding** was based on the TiDIER framework [30]: aims; brief description; mode of delivery; who conducted study/intervention; duration and frequency of contacts; theoretical basis; analysis framework; individual tailoring; modifications/fidelity; collaboration/engagement.

**Outcomes:** how assessed (e.g. mail, face to face); unit of analysis; results summary; conclusion summary; detailed results under each of the main outcome categories (theories; mediating/moderating factors; parent experiences/perceptions; interventions; assessment tools and ‘other’).

### Assessment of risk of bias within studies

For each included study, the risk of bias was assessed independently by two reviewers using domains from one of the following tools as appropriate for the study design (S5 Appendix).

1. RCTs and cluster RCTs: Cochrane risk of bias tool [31].
2. Controlled studies: Cochrane risk of bias tool with additional EPOC terms [32].
3. Cohort/observational studies: ROBINs [33].
4. Qualitative studies: CASP tool [34].
5. Assessment/screening tool accuracy studies: QUADAS checklist [35].

Overall confidence in study findings was assessed using an adaption of the GRADE approach (S6 Appendix). This provides a transparent and systematic method for incorporating information about study limitations (risk of bias), the validity of outcome measures (indirectness/relevance), and the adequacy of the sample (imprecision) in a summary of findings for each study. The overall assessment was conducted by one reviewer (CC), with the first ten assessments checked and discussed with a second reviewer (SB) before completing the remaining assessments. All studies started with an assessment of ‘high’ confidence, and were downgraded one level for serious concerns or two levels for very serious concerns about: study/methodological limitations; indirectness/relevance; and imprecision/adequacy to attain a final assessment of high, moderate, low or very low confidence. Overall confidence across study findings (e.g. consistency and publication bias) was not assessed in this scoping review.

### Data synthesis

Outcome data were synthesized narratively under each of the main outcome categories (theories, risk and protective factors, parents’ views, interventions, assessment tools). Outcomes are summarised only briefly in this scoping review to map and illustrate the breadth of evidence, rather than offer a comprehensive assessment of strengths and limitations of the evidence. Evidence map templates were generated using primary and secondary outcomes specified in the scoping review protocol. Each study providing relevant information was illustrated numerically in the map/table, using a number which corresponds to an allocated study ID number in the Characteristics of Included Studies (COIS) Table (Table 1). The COIS table (Table 1) provides summary study-level information on the country, setting, participant number, parenting stage, parental childhood maltreatment history, study type, study aim, main results and overall confidence in study findings. In subsequent systematic reviews, we will use synthesis methods appropriate for specific study designs and explore the respective evidence and strengths and limitations of the evidence in more depth (S1 Appendix).

**Table 1:** Characteristics of included studies.

Study number/ Study First Author surname/year (Associated refs)* = data extracted for AR [ref]	Country Setting [Study years if reported]	Number of participants (brief description)	Parenting stage	Childhood Trauma History (including proportion with history)	Study type	Study aim (specific)	Briefly describe the main results of the study	Confidence (primary study)
1. Adams 1996 [36]	USA (San Diego, California)	206 parents	Pregnancy	13/89 mothers reported emotional abuse, physical abuse, sexual abuse, neglect, or ‘other‘. (Fathers self-report considered too low to be reliable.)	Cross-sectional	**Screening and uptake of support** (1) to determine the rate at which expectant first-time mothers will identify themselves as mistreated during childhood and, (2) for those who will, the rate at which they will accept offers of support services.	43% expectant mothers responded to the survey. Of these almost 15% reported being abused. Nine of those 13 (69%), as well as 20 of the 76 (26%) who denied abuse, expressed interest in services ‘especially designed for expectant parents who were mistreated as children’.	Very low
2. Ahlfs-Dunn 2015 [37]	USA (Washentaw and Wayne County)	120 ’low income or high risk’ mother-infant dyads (46% African American, mostly unmarried but college educated)	Pregnancy to two years postpartum	Emotional, physical, and sexual abuse, and emotional and physical neglect, childhood intimate partner violence (IPV) exposure, adult IPV.	Prospective cohort	**Investigate intergenerational pathways:** disrupted maternal representations of the child as a mediator of the association between mothers’ histories of interpersonal trauma and their infants’ socio-emotional development at 1 year of age.	There was an indirect effect of maternal childhood interpersonal trauma history on infant attachment security through disrupted prenatal maternal representations of the child.	High
3. Altemeier 1986 [38]	USA (Inner city hospital, 1975–1976)	927 mother-infant dyads (caucasian only, mostly low income, married with less than high school education)	Pregnancy	Physical abuse	Prospective cohort	**Investigate intergenerational pathways:** rates of abuse and characteristics associated with continuity.	Compared to mothers with no abuse history, abused mothers were: more likely to have felt unwanted and unloved as children, to have lower self-images and more isolation, greater stress (many reflecting disturbances in interpersonal relationships). However children were reported to protective services for abuse at the same frequency as control children.	Low
4. Aparicio 2016 [39]	USA (Foster-child transition programme)	6 teen mothers transitioning out of foster care (5 African-American and homeless)	unclear	Physical abuse, sexual abuse, neglect, witness to domestic violence	Qualitative (Interviews)	**Parent strategies to break cycle:** ’How do teen mothers in foster care experience motherhood in terms of working to break the cycle of child abuse and neglect?)	Two themes emerged: (i) treating children well/parenting differently and avoiding the system; and (ii) reducing isolation and enhancing support.	Low
5. Armstrong 1999 [40]	Australia (Brisbane hospital, 1996)	181 ’at risk’ parents (only mother’s outcomes assessed) (6% Indigenous, almost 20% did not complete high school, 40% sole parents).	Birth to 6 weeks postpartum	Childhood abuse’ otherwise unspecified (part of range of inclusion criteria)	Intervention (RCT)	**Home visiting intervention** for ’at risk’ parents	At 6wks pp, women in the intervention group had significant reductions in postnatal depression (primiparous women only), and improvements in their parental role and ability to maintain their own identity. Maternal-child interactions were likely to be more positive, with higher scores related to aspects of the home environment, particularly secure maternal infant-attachment.	Very low
6. Arons 2005 [41]	USA (Massachusetts, Jewish family service)	1 mother-infant dyad receiving therapy	12 months postpartum ongoing	Physical (attempted homicide) and emotional abuse	Case study	**Psychotherapy** aims: the ability to recognize, to name, and to metabolize feelings; the ability to evoke a soothing maternal affect to aid in containment and integration of self-states; and the ability to be aware of and to relate to the partner’s mind.	Detailed vignette concluding: "Mary’s need to defend against the feelings John aroused coupled with her cognitive dysregulation (dissociation and transient thought disorder) had rendered her unable to consistently attend to their relationship. In mother-baby sessions we worked to enhance responsive relating by containing the fear and anger aroused by John’s need for comfort. In individual sessions we explored how Mary’s attachment needs within the transference paralleled those of her son.…Less constricted by her own defensive exclusion of painful affects, Mary developed freer access to her own inner world and to the emotional world of her son. As she began to release John from her malevolent projections and her need to control the fear he aroused, he emerged as a positive force of nature, a baby to be loved and understood."	Moderate
7. Barrett 2009 [42]	USA (Illinois)	494 mothers receiving Temporary Assistance for Needy Families (TANF) benefits participating in waves 1 & 2 of Illinois Families study (83% African American and 11% Hispanic, 68% completed high school, 17% married/partner, 30% prior CPS involvement)	Children 3 years of age or younger	Child sexual abuse (CSA) and 5 other forms of childhood adversity (childhood physical abuse, perceiving that one had been neglected in childhood, observing domestic violence, childhood poverty, and living apart from one or both parents for all or part of childhood).	Cross-sectional study within longitudinal cohort.	**Investigate intergenerational pathways:** explore the association between CSA and adulthood parental stress, parental warmth, use of nonviolent discipline strategies, psychological aggression, and use of physical punishment. Additionally, whether other forms of childhood adversity altered this relationship.	CSA survivors reported significantly lower rates of parental warmth, higher rates of psychological aggression, and more frequent use of corporal punishment than mothers who had not experienced childhood sexual abuse. These effects, however, were non-significant when sociodemographic factors and other forms of childhood adversity were considered, suggesting that CSA may not be as salient a predictor of certain parenting practices than other forms of childhood adversity.	High
8a. Bartlett 2015 [43] (8b. Bartlett 2012) [44]	USA (Massachusetts, 2008–2010)	447 mothers participating in Home Visiting Program evaluation (ethnically diverse, 56% receiving welfare).	Pregnancy (64%) to postpartum (36%) (child’s age unclear). This study uses RCT data collected at enrolment) and one year later.	Physical abuse, sexual abuse, neglect	Prospective cohort (RCT participation as a control variable)	**Investigate intergenerational pathways:** examine whether positive care in childhood, social support while parenting, and older maternal age at birth moderate the association between young mothers’ childhood history of abuse and neglect and neglect of their own infants, and maternal lack of empathy as a correlate.	Approximately 77% of maltreated mothers broke the cycle with their infants (<30 months). Maternal age moderated the relation between a maternal history of neglect and infant neglect, and social support moderated the relation between childhood neglect and maternal empathy. Neglected mothers had considerably higher levels of parenting empathy when they had frequent access to social support than when they had less frequent support, whereas the protective effect of social support was not nearly as strong for non-maltreated mothers.	Moderate
9. Baumgardner 2007 [45]	USA (Baltimore Maryland, 1997 ongoing)	141 women (African American, under 18 years of age, primiparous, low income (eligible for the Special Supplemental Nutrition Program for Women, Infants, and Children—WIC), and residing within three-generation households.	Shortly after birth and this study reports followup at 24 months postpartum.	Physical and sexual abuse (38% reported abuse)	Cross-sectional study within longitudinal cohort.	**Investigate intergenerational pathways:** view of three generations examining a history of child abuse within the larger context of relationship quality to consider: (1) How does a history of abuse relate to the early parent-child relationship? (2) whether a history of abuse correlates with problems in the relationships between generations (3) does a more positive relationship with one’s own mother act as a buffer? (4) whether there are components of the intergenerational relationship, beyond attachment, that are affected by a history of childhood abuse, and (5) whether different types of abuse contribute to problems in the early mother-baby relationship.	Grandmother-adolescent mother relationship did show some moderating effect, in the presence of abuse, on parent-child interaction. Even in the presence of a history of childhood abuse, when grandmother-adolescent mother relationship quality is good and characterized by mutuality, autonomy, and comfort with negotiating disagreement, maternal behaviours promoting attachment processes and infant social competence are high.	Moderate
10a. Baydar 2003 [46] (10b. Webster Stratton 1998b [47]; 10c. Webster-Stratton 2001 [48])	USA (Puget Sound area Head Start centers, 1993 to 1997)	426 parents participating in Incredible Years Parenting Training Program (60% caucasian).	Pregnancy with followup after 6–12 week intervention	Measures of “harsh/ negative, supportive/positive, and inconsistent/in- effective parenting"	RCT/SEM	**Parenting intervention**: Evaluation of ‘Incredible Years’ program compared to Head Start’ curriculum to improve understanding of the way some psychological risk factors influence mothers’ parenting, mothers’ participation in parent training programs, and their ability to benefit from the parenting program.	Parent engagement training was associated with improved parenting in a dose-response fashion. Mothers with mental health risk factors (i.e., depression, anger, history of abuse as a child, and substance abuse) exhibited poorer parenting than mothers without these risk factors. However, mothers with risk factors were engaged in and benefited from the parenting training program at levels that were comparable to mothers without these risk factors.	Moderate
11a. Berlin 2011 [49] (11b. Appleyard 2011*) [50]	USA (Prenatal care clinics in a small southeastern city and its surrounding county)	499 women (diverse ethnicity, most have partner, high proportion less than high school)	Pregnancy to two years postpartum	Physical abuse, sexual abuse and neglect (Parent-Child Conflict Tactics Scale)	Prospective cohort	**Investigate intergenerational pathways:** to examine associations between mothers’ childhood physical abuse and neglect and their child’s victimization during the first two years of life (Berlin 2011) and maternal substance use as a mediator (Appleyard 2011).	Mothers’ childhood physical abuse—but not neglect—directly predicted offspring victimization. This association was mediated by mothers’ social isolation, aggressive response biases (Berlin 2011). Maternal substance use was significantly associated with history of physical and sexual abuse, and mediated the mediated the pathway to offspring victimization (Appleyard 2011).	High
12. Blackmore 2016 [9]	USA (Hospital obstetric clinic, 2007–2012)	358 women (ethnically diverse, Mostly high school educated, 70% receiving Medicaid, 55% sole parents).	18 and 32 weeks of pregnancy	Physical and sexual abuse, neglect.	Prospective cohort (longitudinal)	**Investigate intergenerational pathways:** trauma and trauma severity and adverse obstetric outcomes (lower birthweight, earlier gestational age, or delivery complications.)	Childhood trauma exposure increased vulnerability for low birthweight delivery associated with prenatal mood disturbance.	High
13a. Blalock 2011 [51] (13b. Blalock 2013*)[52]	USA (Texas, Houston metropolitan area, 2005–2008)	201 women smoking in early pregnancy (Over 60% African-American or Hispanic, 34% less than high school, 65% receiving Medicaid, 48% sole parents).	Pregnancy	Emotional, sexual, and physical abuse and emotional and physical neglect categorised using cut scores to delineate none to minimal, low to moderate, moderate to severe, and severe to extreme levels of maltreatment. 59% sample experienced maltreatment.	RCT	**Smoking cessation intervention:** examines link between childhood trauma and smoking, and mediating role of depression (Blalock 2011); and whether childhood trauma moderated the treatment effect from a smoking cessation trial (Blalock 2013).	Moderate to extreme levels of childhood trauma were significantly related to smoking dependence and were partially mediated by depressive symptoms (Blalock 2011). There was a dose response association of treatment on depression outcome through 6 months postpartum; those with increasing amounts of childhood trauma benefitted more from CBASP, compared to those in the HW condition. Childhood trauma did not moderate the treatment effect on smoking abstinence, although increasing amounts of trauma were associated with reduced likelihood of abstinence at 6 months post treatment (Blalock 2013).	Moderate
14. Bouvette-Turcot 2015 [53]	Canada (Montreal (Quebec) and Hamilton (Ontario) antenatal clinics)	154 mother-term infant dyads (89% caucasian).	Pregnancy to 36 months postpartum	Emotional, physical and sexual abuse, emotional and physical neglect.	Prospective cohort	**Investigate intergenerational pathways:** relation between maternal adversity and NE/BR in the child, and if the effect of maternal childhood adversity is moderated by child 5-HTTLPR genotype.	There was a significant interaction effect of maternal childhood adversity and offspring 5-HTTLPR genotype on child negative emotionality/behavioural dysregulation.	Moderate
15a. Brand 2010 [54] (15b. Cammack 2016* [55], 15c. Juul 2016* [56])	USA (Atlanta, Georgia Emory Women’s Mental Health Program, 2002–2011)	126–255 women participating in mental health program, predominantly caucasian (94%), married (95%) and college-educated (82%).	Pregnancy to 6 months postpartum	Emotional, physical, sexual abuse and emotional and physical neglect. 30% sample experienced trauma.	Cross-sectional study within longitudinal cohort/Test retest survey.	**Investigate intergenerational pathways) (1)** (investigate the association between maternal history of child abuse and maternal cortisol levels in a clinical sample of postpartum women, and to explore whether depressive symptoms and stressful life events, as well as comorbid PTSD moderated this relationship, and transgenerational effects on the infant (Brand 2010). **(2)** Examine the stability of responses to the CTQ at two pregnancy timepoint (Cammack 2016) and (3) relationship between maternal history to differences in maternal affect and HPA axis functioning (Juul 2016).	Maternal childhood abuse was associated with steeper declines in cortisol in the mothers, and lower baseline cortisol in their infants. Comorbid maternal PTSD, current maternal depressive symptoms, and recent life stressors significantly moderated maternal cortisol change. Maternal abuse history was associated with increases in cortisol levels in those mothers who experienced these additional stressors. Similarly, a history of early maternal abuse and comorbid PTSD was associated with greater increases in infant cortisol levels (Brand 2010). CTQ response reliability was generally at least moderate, indicating consistent reporting across two time-points (Cammack 2010). Childhood maltreatment history predicted increased neutral affect and decreased mean cortisol in the mothers and that cortisol mediated the association between trauma history and maternal affect. Maternal depression was not associated with affective measures or cortisol (Juul 2016).	Low
16. Bysom 2000 [57]	USA	29 mothers who had experienced childhood abuse or witnessed IPV (mean age 40, predominantly college-educated and married).	Unclear	Physical abuse or witnessing IPV before age 13 (whole sample).	Cross-sectional study (mixed quantitative/qualitative)	**Investigate intergenerational pathways: to** identify types of social support utilized by resilient individuals from abusive backgrounds; and to explore the relationship between perceived social support and the ability to interrupt the cycle of violence.	No one type of social support was identified as being more prevalent than another. There was a positive relationship between perception of one’s own abuse after the age of 13 and type of support labelled as belonging social support. Similarly, there was a positive correlation between experienced abuse after the age of 13 and belonging and self-esteem support. There was a negative correlation between perception and experience of one’s own abuse prior to age 13 and the presence of a caring adult in childhood and a negative correlation between the perception and experience of one’s own abuse after age 13 and the presence of a caring adult and friend in childhood. Two thirds of the participants were affirmative in their belief that social support was helpful in breaking the cycle of violence. Narrative themes of social support, internal aspects of self and spirituality emerged as factors which played a role in breaking the cycle of violence. Awareness of alternatives and non-judgmental support were identified as aspects of social support which were deemed helpful.	Very low
17. Caliso 1992 [58]	USA (Division of Youth and Family Services and the Correctional Institution for Women)	90 mothers (30 perpetuating abuse, 30 discontinuing abuse and 30 with no abuse history (matched on socio-demographic characteristics: mean age 30, ethnically diverse, mean education 12 years, 30% married).	Unclear	Physical abuse (66% sample)	Cross-sectional study	**Investigate intergenerational pathways:** To determine the effect of a childhood history of abuse on adult child abuse potential.	Conflict Tactics Scale (CTS) verbal and violence scales were higher for the abusive and non-abusive mothers with a childhood history of abuse. Child Abuse Potential (CAP) scores distinguished between all three study groups. However, only the rigidity and unhappiness factors discriminated between abusive and non-abusive mothers with a childhood history of abuse. Non-abusive mothers with a childhood history of abuse were less rigid in their child expectations and were happier in their interpersonal relationships than abusive mothers with a childhood history of abuse.	Low
18. Chemtob 2011 [59]	USA (child welfare agencies in New York)	127 mothers receiving preventive CW services with PTSD score >15. (63% Latina & 19% African American, 41% sole parents).	Preschool children	Any maternal trauma—including adult sexual assault, war, illness, imprisonment.	Cross-sectional study	**Investigate intergenerational pathways:** (**1)** trauma exposure rates among mothers and children receiving preventive services screened by the preventive services partners; (**2**) maternal PTSD and depression symptoms among the mothers in preventive services screened; and (3) ethnic differences in trauma exposure and trauma-related symptoms	There were high levels of trauma exposure among screened mothers (92%) and their young children (92%). 54.3% mothers met probable criteria for posttraumatic stress disorder (PTSD) or depression (61.7%). Nearly half (48.8%) met criteria for co-morbid PTSD and depression. Most were not receiving mental health services. Latina women had significantly more severe PTSD symptoms than African American women. Case planners reported that the screening process was useful and feasible.	High
19a. Chung 2009 [60] (19b. Chung 2008* [61], 19c. Chung 2006 [62], 19d. Chung 2004 [63], 19e.Culhane 2001 [64])	USA (community health centres in Philadelphia, Pennsylvania, 2000–2002)	1265–1476 women completing pre and postnatal interviews (predominantly African-American/Latina and low income, 40% did not complete high school, 78% sole parents).	Pregnancy to 11 months postpartum	Physical abuse, sexual abuse, verbal hostility, domestic violence, witnessing or knowing a victim of a shooting (ACE). (76% reported at least 1 ACE and 51% reported at least 2.)	Prospective cohort	**Investigate intergenerational pathways**: (**1**) to assess associations among maternal childhood experiences and subsequent parenting attitudes and Infant spanking (IS), and to determine if parenting attitudes mediate (Chung 2009) (**2**) association between adverse childhood experiences (ACEs), positive influences in childhood (PICs), and depressive symptoms among low-income pregnant women (Chung 2008).	(**1**) Mothers exposed to childhood physical abuse and verbal hostility were more likely to report IS use than those not exposed. In the adjusted analyses, maternal exposure to physical abuse, other ACEs, and valuing CP were independently associated with IS use. Attitudes that value CP did not mediate these associations (Chung 2009). (**2**) For each ACE, affected women were more likely to have depressive symptoms than their counterparts. There was a dose-response effect in that a higher number of ACEs was associated with a higher likelihood of having depressive symptoms. PICs, on the other hand, were associated with a lower likelihood of having risk. Efforts to prevent ACEs and to promote PICs might help reduce the risk of depressive symptoms and their associated problems in adulthood (Chung 2008).	Moderate
20. Coles 2009 [65]	Australia (Melbourne Metropolitan area, 2005)	11 mothers who had breastfed their baby.	Less than 2 years postpartum	Sexual abuse (whole sample).	Qualitative (Interviews)	To **explore the experience of successful breastfeeding** with mothers with a history of CSA.	Four key themes are identified: enhancement of the mother—baby relationship, validation of the maternal body, splitting of the breasts’ dual role as maternal and sexual objects, and exposure and control when breastfeeding in public.	Moderate
21. Conger 2013 [66]	USA (rural counties in north central Iowa, 1994 Generation (G1) and 2005 (G2)).	290 parents (G2) (120 males, 170 females) who had a G3 child eligible for participation by 2005.	Min 18 months, mean 2.31 years postpartum	Direct observation of G1 mother hostility (angry or rejecting behaviour), angry coercion (demanding, stubborn, coercive), physical attacks (hitting, pushing, pinching, etc.), and antisocial behaviour (self-centred, immature, insensitive) behaviour toward the G2 parent during adolescence.	Prospective cohort	**Investigate intergenerational pathways:** dimensions of adult romantic relationships that hold promise for reducing continuity in harsh parenting.	Romantic partner warmth and positive communication with G2 were associated with less G2 harsh parenting toward G3 (a direct effect) and when these partner behaviours were high, there was no evidence of intergenerational continuity from G1 to G2 harsh parenting. When the partner was low on warmth and communication, intergenerational continuity in harsh parenting significantly increased. G1 harsh parenting slightly decreased the likelihood that G2 would select a positive spouse.	High
22. Dijlska 1995 [67]	Netherlands (1994)	2 mothers	One with an 18month old and the other with a 6yo and 9yo child.	Physical abuse, sexual abuse, neglect	Qualitative/Case study	**Parent strategies to break cycle:** how intergenerational patterns of violence are transformed and broken.	Invisibility of former abuse in adult life and limited ability to speak freely about the meaning of this. Discusses process orientated trauma therapy and transformations due to time and developmental stages/triggers—such as having a child and changing work challenges. Suggests triggers can be viewed as ticking time bombs but also as opportunities for working through certain aspects of trauma.	Low
23a. Dixon 2005 [68] (23b. Dixon 2005b*[69], 23c. Dixon 2009*[70])	UK (Southend on Sea, Essex, England, 1995–1998)	4351 parents (majority caucasian)	During first 13 months postpartum	Physical and/or sexual abuse in their own childhood (<16 years), (135/4351 parents).	Prospective cohort	**Investigate intergenerational pathways (1)** to explore risk factors, parental attitudes and behaviour implicated in the intergenerational cycle of maltreatment (Dixon 2005); (**2)**: explore the mediating properties of secure relationships and their interplay with risk factors in the intergenerational cycle of maltreatment (Dixon 2005b) and; **(3)** investigate factors associated with both the continuation and discontinuation of the intergenerational transmission of child maltreatment (Dixon 2009).	Within 13 months after birth, 6.7% AP families were referred for maltreating their own child in comparison to.4% NAP families. AP families had significantly higher numbers of risk factors. Mediational analysis found that intergenerational continuity of child maltreatment was explained to a larger extent (62% of the total effect) by the presence of poor parenting styles together with the three significant risk factors (parenting under 21 years, history of mental illness or depression, residing with a violent adult). The three risk factors alone were less explanatory (53% of the total effect) (Dixon 2005, 2005b). Protective factors of financial solvency and social support distinguished Cycle Breakers from Maintainers and Initiators. Therefore, it is the presence of protective factors that distinguish Cycle Breakers from families who were referred to Child Protection professionals in the first year after birth (Dixon 2009).	Moderate
24. Draucker 2011 [71]	USA (metropolitan Akron, OH)	95 men and women (50% African-American, 68% employed).	unclear	Sexual abuse (mostly in context of caregiving relationship). Whole sample.	Qualitative (Interviews)	To **describe, explain, and predict women’s and men’s responses to sexual violence** throughout their lives and construct a healing model.	The CSA Healing Model is a model that represents the multifaceted and dynamic process of healing from CSA over the lifespan. The model does not focus on discrete variables associated with positive or negative coping but rather captures some complex healing processes that culminate in the experience of laying claim to one’s life. The model includes four stages of healing, five domains of functioning, and six enabling factors that facilitate movement from one stage to the next. The model indicates that clinicians should focus on how clients might move from grappling with the meaning of the CSA, to figuring out its meaning, to tackling its effects, and ultimately, to laying claim to their lives, and they should be ready to discuss healing in any one of several domains. Because parenting was of great concern to most participants, it should routinely be addressed as a therapeutic issue. The model suggests that parenting should not be considered a dichotomous factor; that is, one either abuses or one nurtures one’s children. Rather, the processes of wishing to stop the cycle of abuse and attempting to stop the cycle of abuse were critical steps that need to be acknowledged and fostered.	Moderate
25a. Egeland 1996 [72] (25b. Egeland 1988 [73], 25c. Bosquet 2016 [74])	USA (Maternal and Infant Care Clinics, Minneapolis Health Department, from 1975)	24, 30 (Egeland) and 187 (Bosquet 2016) mother-infant dyads (80% caucasian, 40% had not completed high school, 62% sole parents).	Pregnancy (recruitment) to 48–54 months postpartum	Sexual abuse, physical abuse or neglect (Edgeland -whole sample, Bosquet includes women with and without abuse history).	Prospective cohort	**Investigate intergenerational pathways** (**1)**: to determine whether two groups of mothers, those who broke the cycle of abuse compared to those who did not, differ on dissociative process and symptomatology (Egeland 1996); **(2**) contrast the incidence of supportive relationships experienced by mothers who continued the cycle by abusing their own child versus those who broke the cycle and provided the child with adequate care (Egeland 1988); and **(3)** examine whether a maternal history of maltreatment in childhood has a detrimental impact on young children’s mental health and to test theoretically and empirically informed pathways by which maternal history may influence child mental health (Bosquet 2016).	**(1**) Mothers who were abused and are abusing their children were rated higher on idealization, inconsistency, and escapism in their description of their childhood and they scored higher on the Dissociative Experience Scale compared to mothers who broke the cycle. Mothers who were abused and abused their children recalled the care they received as children in a fragmented and disconnected fashion whereas those who broke the cycle integrated their abusive experience into a more coherent view of self. Even after partialling out the effects of IQ, large differences were found indicating that dissociative process plays a part in the transmission of maltreatment across generations (Egeland 1996). **(2)** Abused mothers who were able to break the abusive cycle were significantly more likely to have received emotional support from a nonabusive adult during childhood, participated in therapy during any period of their lives, and to have had a nonabusive and more stable, emotionally supportive, and satisfying relationship with a mate. Abused mothers who re-enacted their maltreatment with their own children experienced significantly more life stress and were more anxious, dependent, immature, and depressed (Egeland 1988). **(3)** Maltreated mothers experienced greater stress and diminished social support, and their children were more likely to be maltreated across early childhood. By age 7, children of maltreated mothers were at increased risk for clinically significant emotional and behavioral problems. A path analysis model showed mediation of the effects of maternal childhood maltreatment history on child symptoms, with specific effects significant for child maltreatment (Bosquet 2016).	Very low
26. Ensink 2016 [75]	Canada (community)	88 women with uncomplicated pregnancies (All caucasian, mostly college-educated, 66% married).	Pregnancy to 16 months postpartum	Physical, sexual, or emotional abuse (30%) (AAI).	Longitudinal study/ prospective cohort	**Investigate intergenerational pathways:** from mothers’ RF regarding attachment through parenting to infant attachment.	Mothers’ mentalization regarding their own early attachment relationships was associated with later parenting and infant attachment. Negative parenting behaviours explained the link between mothers’ RF about their own attachment relationships and infant attachment disorganization.	Low
27. Esaki 2008 [76]	USA (East (EA), Midwest (MW), and South (SO) sites, 1991–2004)	477 mothers categorised as ’high risk’ (80% ’non-white', lean less than high school education, 75% sole parents, 49% reported IPV)	1, 4, 6 and 8 years postpartum	Physical and sexual abuse	Longitudinal/ prospective cohort	**Investigate intergenerational pathways:** (**1)** the relationship between experiences of multiple types of maternal childhood abuse and frequency of perpetration of child maltreatment in adulthood. **(2)** whether parenting attitude predicts child maltreatment and mediates the relationship between maltreatment history and maltreating one’s own children. **(3)** the moderating effect of social support on the relationship between maternal childhood abuse and parenting attitude. **(4)** the role of social support in moderating the relationship experiences of maltreatment and perpetration of child maltreatment.	Multiple types of maternal childhood abuse were significantly associated with increased frequency of perpetration of child maltreatment in adulthood. No mediating role of parenting attitude between maternal childhood abuse and perpetration of child maltreatment in adulthood or for the moderating role of social support on this pathway were found.	Moderate
28a.Fornburg 1996 [77] (28b. Gara 2000) [78]	USA (inner-city community mental health clinics, early prevention/baby nutrition programs and an early intervention program, New Jersey).	60 first time mothers with and without abuse (matched case control) (60% African-American, 24% Hispanic)	Less than 6 months postpartum	Physical abuse (AEIII-SD). (50%)	Prospective cohort	**Investigate intergenerational pathways:** to identify changes in mothers’ perceptions of their baby, in terms of negative and positive attributions, over the course of the first year of the baby’s life.	The abused mothers perceived their baby in significantly less positive terms at age 12 months than they had at age 6 months of the baby.	Very Low
29a.Grote 2012 [20] (29b. Grote 2009 [79]; 29c. Grote 2014 [80]; 29d. Grote 2015 [81])	USA (large public obstetrics and gynaecology clinic in Pittsburgh, Pennsylvania)	53 women experiencing prenatal depression (70% African-American, >80% completed high school, 50% sole parents).	Pregnancy (T1) to 6 months postpartum (T3).	Emotional abuse, physical abuse, sexual abuse, emotional neglect, and physical neglect (CTQ).	Intervention/RCT	**Psychological intervention** (compare UC to brief IPT (IPT-B) plus IPT maintenance.	Trauma exposure did not moderate changes in symptoms and functioning over time for women in UC versus IPT-B, suggesting that IPT including maintenance sessions is a reasonable approach to treating depression in this population. Within the IPT-B group, women with more versus less trauma exposure had greater depression severity and poorer outcomes at 3-month post baseline. At 6-month postpartum, they had outcomes indicating remission in depression and functioning, but also had more residual depressive symptoms than those with less trauma exposure, suggesting longer periods of support may be required for women with trauma experiences.	Moderate
30. Harmer 1999 [82]	Australia (substance use treatment centre in the Australian Capital Territory, 1995–1996)	46 mothers recovering from addiction (predominantly caucasian (3 Indigenous), low-middle income, all sole parents)	6 months to 12 years age	Negative home environment/neglect, sexual abuse and punishment (62% had experienced abuse)	Cross-sectional study	**Investigate intergenerational pathways:** association between mothers’ aversive childhood experiences, their subsequent social support in adulthood, psychological distress, parenting stress, and problematic parenting behaviours; and moderating effect of social support.	Mothers in this substance use treatment centre reported very high levels of aversive childhood experiences, psychological distress, parenting stress and use of problematic parenting behaviours along with lower levels of social support. Higher levels of neglect and growing up in a negative home environment were significantly correlated with lower levels of social support from the family, higher levels of distress and parenting stress, and greater use of problematic parenting behaviours.	Low
31. Herronkohl 2013 [83]	USA (child welfare and community centres, 1975–2010)	268 parents (78% caucasian)	unclear	Physical abuse (severe ’Harsh physical discipline')	Longitudinal/cross-sectional study	**Investigate intergenerational pathways:** examine evidence of the continuity in abusive discipline across two generations (G1 and G2) and the role of Safe, Stable, and Nurturing Relationships (SSNRs) as protective factors.	Significant predictive association between physical discipline by G1 and G2 parents, after accounting for childhood socioeconomic status and gender. While being physically disciplined as a child was inversely related to reports of having had a caring relationship with one’s mother, only care and support from one’s father predicted a lower risk of harsh physical discipline by G2s. None of the SSNR variables moderated the effect of G1 discipline on G2 discipline. A case-level examination of the abusive histories of G2 harsh discipliners found they had in some cases been exposed to physical and emotional abuse by multiple caregivers and by adult partners.	Moderate
32. Hooper 2004 [84]	UK (North Yorkshire)	24 mothers with CSA.	unclear	Sexual abuse (whole sample). Many had also experienced other forms of abuse.	Qualitative/interviews	**Experience of services**: ways in which their good intentions towards their children could be undermined by their experience and environment, with the aim of identifying how this group of mothers could be better supported.	Many issues may affect survivors’ well-being and access to social support and hence their ability to care effectively for their children. For example the impacts of survivors’ issues around attachment, and the impact on children of deterioration in their mothers’ mental health when appropriate services are not available. Ways of supporting both survivors and their children involve greater collective responsibility for children, effective collaboration between mental health services and child-care services, and professional responses which take account of contextual issues.	Moderate
33. Hunter 1979 [85]	USA (Special Care Nursery)	40 parents of infants born preterm, with a history of childhood abuse and no perpetuation of violence by 12 months postpartum (>60% married).	After birth to 12 months postpartum	Mistreated’ (63% mothers, 55% fathers)	Prospective cohort	**Investigate intergenerational pathways:** Examine protective factors in ’non-repeating’ families	The mechanisms for change included reliance on a broad network of resources, a degree of self-differentiation, an attitude of realistic optimism, and the ability to marshal extra resources to meet crisis situations.	Very low
34. Iyengar 2014 [86]	USA	47 first-time mothers (ethnically diverse, majority married).	Pregnancy to 11 months postpartum	Unresolved or resolved trauma and AAI (secure or insecure).	Prospective cohort	**Investigate intergenerational pathways**: to examine; the process of reorganization on the transmission of attachment across generations; associations between unresolved trauma and mother-child attachment, while testing whether reorganization is associated with more secure child attachment.	Mothers with unresolved trauma had insecure attachment themselves and were more likely to have infants within secure attachment. However, the one exception was that all of the mothers with unresolved trauma who were reorganizing toward secure attachment had infants with secure attachment.	Low
35. Kunseler 2016 [87]	Netherlands (prenatal clinics)	243 first time mothers, 101 ’at risk’ who reported experiences with youth care or with a psychiatrist or psychologist before the age of 18, and 142 comparison group. (73% Dutch, 58% university educated)	Pregnancy to one year postpartum.	Physical maltreatment, sexual abuse, bizarre punishments of the child, parents’ attempts of suicide, or other frightening behaviours exhibited by parents in presence of the child (AAI). (25%)	Cross-sectional study	**Investigate intergenerational pathways**: whether women who report abuse in childhood adapt in less resilient ways to challenges to their sense of parenting competence due to infant difficult behaviour than women who report no childhood abuse.	Pregnant women who reported childhood abuse decreased more in Parenting Self Efficacy (PSE) in response to the difficult-to-soothe infant (i.e., failure condition) than pregnant women who reported no abuse, whereas no differences were found in women’s adjustment of PSE to the easy-to-soothe infant (i.e., success condition) or with respect to PSE at baseline. Effects did not vary according to resolution of trauma.	Moderate
36a. Lancaster 2007 [88] (36b. Hill 2000 [89], 36c. Hill 2001 [90])	UK	192	Unclear	Sexual abuse, physical abuse, psychological abuse, antipathy and neglect.	cross-sectional study	**Screening/assessment tool evaluation**	The discriminative ability of PBI care scores to predict measures of neglect in the CECA were moderate to high, and the addition of paternal scores did not add to the prediction from maternal scores. Shortened forms of the PBI maternal care scales provided comparable predictions to those from the full scale, particularly three items from the maternal care scale, identified by logistic regression.	Low
37. Leifer 1990 [91]	USA (early-intervention program at the Child and Adolescent Psychiatric Clinic, Chicago)	1 mother-infant dyad receiving therapy (16 yo African-American living with her mother, living on aid and did not complete high school).	4 months pp onwards.	Physical and sexual abuse and neglect.	Case study	**Psychotherapy description**: to illustrate the diagnostic and therapeutic issues in the course of an intervention designed to prevent intergenerational abuse.	Drawing upon an ecological, transactional model of development, the case study utilized a multimethod, longitudinal approach to assess the mother’s history and current psychosocial functioning, the infant’s developmental competence and attachment status, patterns of mother-infant interaction and components of the family’s social ecology. The treatment involved two weekly therapy sessions; one, an individual session for the mother and the other, a session in which mother and infant were seen together. The findings at the one-year evaluation showed improved maternal psychosocial functioning, the infant’s shift from an insecure to a secure attachment classification and improved patterns of mother-infant interactions.	Low
38. Libby 2008 [92]	USA (Southwest and Northern Plains Indian tribes, 1997).	2221 Indigenous parents (Almost 50% reported financial strain, fathers reported lower social support scales and higher rates of alcohol abuse than mothers)	Up to 13 years of age.	Physical and sexual abuse prior to 13 years of age.	Cross-sectional study	**Investigate intergenerational pathways**: To examine the relationship of childhood physical and sexual abuse with reported parenting satisfaction and parenting role impairment later in life among American Indians (AIs).	Only substance use, not depression, mediated the relationship between childhood abuse and parenting outcomes. Instrumental and perceived social support significantly enhanced parenting satisfaction; negative social support reduced satisfaction and increased the likelihood of parenting role impairment. Exposure to parental violence while growing up had deleterious effects on parenting outcomes. Mothers and fathers did not differ significantly in the relation of childhood abuse experience and later parenting outcomes.	Moderate
39. Lieberman 2005 [93]	USA (child–parent psychotherapy)	Ethnically and socioeconomically diverse sample.	Birth to 6 years	Unclear	Qualitative/Clinical record review (AAI transcripts)	**Parent strategies to break cycle**: examine parental narratives in assessment instruments and clinical notes reflecting on their early years, their relationships with their parents; their thoughts on how these experiences influenced their hopes for their children’s future, to identify early experiences of love, care, and nurturing that might stand out as sources of strength in the parents’ sense of themselves and ability to care for their children.	Authors propose that angels in the nursery—care-receiving experiences characterized by intense shared affect between parent and child in which the child feels nearly perfectly understood, accepted, and loved—provide the child with a core sense of security and self-worth that can be drawn upon when the child becomes a parent to interrupt the cycle of maltreatment. Uncovering angels as growth-promoting forces in the lives of traumatized parents is as vital to the work of psychotherapy as is the interpretation and exorcizing of ghosts.	Very low
40a. Lyons-Ruth 1996 [94] (40b. Lyons-Ruth 1990 [95])	USA	45 mothers (low-income,	After birth to 18 months postpartum	Experiences of witnessing violence, neglect, or physical or sexual abuse (Adult Attachment Interview) (47%).	Longitudinal/prospective cohort	**Investigate intergenerational pathways**: (associations between childhood trauma and rates of disorganized infant attachment strategies as well as less involved and more hostile maternal caregiving. and mediating role of the continued presence of trauma-related symptoms in adulthood, such as dissociative or post-traumatic symptoms, unresolved trauma on the caregiving and attachment systems.)	A history of physical abuse was associated with increased hostile-intrusive behaviour toward the infant, increased infant negative affect, and a decreased tendency to act on trauma-related symptoms. A history of sexual abuse was associated with decreased involvement with the infant, more restricted maternal affect, and more active reporting of trauma-related symptoms. Infants of mothers who had experienced childhood violence or abuse were not more likely to display insecure attachment strategies than infants of mothers who had not experienced trauma. However, the form of insecure behaviour was significantly different. Insecure infants of violence-exposed mothers displayed predominantly disorganized strategies, whereas insecure infants of mothers with benign childhoods or neglect only displayed predominantly avoidant strategies.	Low
41a. Madigan 2016 [96] (41b. Madigan 2015*[97])	Canada (Young Parent Resource Centre in large metropolitan children’s hospital)	32 adolescent (12–18 yrs) women enrolled in intervention study (49% African-American, 29% caucasian, 13% Hispanic; mean 10 years school; 89% sole parents; 99% living ’below poverty threshold',)	Pregnancy to 12 months postpartum	Emotional Abuse, Physical Abuse, Sexual Abuse, Physical Neglect, and Emotional Neglect (CTQ/AAI).	Prospective cohort	**Screening/assessment tool evaluation:** Investigate stability of ’unresolved’ (AAI) classification by investigating its stability, as well as exploring predictors of initial levels and rates of change in Unresolved loss and trauma scales across three time points during the developmental transition to parenthood, in a high-risk sample of adolescent mothers.	There is significant stability for the Unresolved classification over the transition to parenthood: Adolescents who were Unresolved prenatally were 8 and 18 times more likely to be classified as Unresolved when their infants were 6 and 12 months old, respectively. On average, there was a steady linear decline in Unresolved loss scores over time, with a rate of change of 27% from the prenatal to 12 months postpartum assessments. There were also significant individual differences in this rate of change. Physical abuse was associated with higher levels of Unresolved loss at the prenatal assessment, and preoccupied attachment attenuated the likelihood of a decline over time in Unresolved loss scores. There was no significant mean rate of change for Unresolved trauma; however, there was considerable variability in scores, with some individuals increasing and others decreasing. Dismissing classifications and a history of sexual abuse were associated with higher levels of Unresolved trauma at the prenatal assessment, and severity of physical abuse was associated with increasing scores of Unresolved trauma over time.	Low
42a. McWey 2013 [98] (42b. McWey 2011 [99])	USA	24 parents notified to CPS (Caucasian (11), African American (10) and Hispanic (3); majority sole parents).	Unclear	Childhood maltreatment (100%).	Qualitative/Interviews	**To examine intergenerational patterns of maltreatment** and understand: how parents at-risk of losing their children because of maltreatment make connections between their own experiences of maltreatment and their children’s experiences of maltreatment; how they desire to parent differently and / or similarly to their own parents; and how these beliefs are reflected in their behaviours with their children.	Three major categories were identified: patterns, beliefs, and behaviours. A majority of the parents stated that they recognized intergenerational patterns, most expressed that they wanted to be different from their own parents, yet many described parenting actions that were ‘‘destructive.”	Moderate
43. Mohler 2001 [100]	Germany (Parent–Infant Programme at Heidelberg University Clinic)	1 (case study)	8 weeks postpartum	Physical abuse	Qualitative/case study	**Parent strategies to break cycle:** to describe the reactions, attitudes and interactional characteristics of a young mother with a history of abuse, illustrating projective mechanisms in the transmission of a ‘potential for violence’.	Maternal perception of her infant was distorted to the extent that the mother was re-experiencing encounters with her own intrusive and traumatizing mother in the face of her screaming child. She also perceived the infant’s motor impulses as physical attacks on herself and expressed intense anxieties about her daughter’s future aggressive potential. She tried to ward off these anxieties by employing a rigid scheme of rules and obsessively controlling the father’s and grandmother’s interaction with the child. For the success of therapy in this case, ‘containment’ of Mrs L’s needs turned out to be important. However, this also underlined the need for including the partner in the therapy in order to make the abused woman’s striving for acceptance, her narcissistic vulnerability and her mistrust of relationships understandable to the husband.	Moderate
44. Monaghan-Blout 1999 [101]	USA (Massachusetts branch of Parents Anonymous, 1996–1998)	8 mothers who viewed some aspect of their parenting to be harmful (mostly caucasian and married; 3 did not complete high school; most depressed).	5 parents between 4 months to 3 years and 3 parents with children under 6	Emotional abuse, physical abuse, sexual abuse, physical and emotional neglect, witnessing of the physical abuse of siblings (100%).	Qualitative (Interviews)	**Parent strategies to break cycle**: investigate the process of resisting the legacy of intergenerational maltreatment as well as explore the ways in which helpers help or fail to help these families as they struggle with the challenges of parenting.	Results suggest that some of the difficulties in helping at risk or maltreating parents may come from an insufficient respect for how the themes developed through a childhood of maltreatment interfere with the establishment of a positive relationship. Key qualities in building a helping alliance were described by the participants, and the good match between client needs and a self/mutual help model were noted. Important aspects of resilient outcomes involved childhood sibling bonds and the successful negotiation of earlier critical events.	Moderate
45a. Montgomery 2015 [102] (45b. Montgomery 2015b [103])	UK (maternity service in South England, 2008–2011)	9 mothers (caucasian, married).	9 weeks to 28 years postpartum	sexual abuse <16yrs	Qualitative (Interviews)	**Experience of services**: (1) Explore maternity care experiences of women who were sexually abused in childhood that demonstrate ways that maternity care can be reminiscent of abuse. (2) Inform practice by exploring the impact that childhood sexual abuse has on the maternity care experiences of adult women.	The main themes identified were: women’s narratives of self, women’s narratives of relationship, women’s narratives of context and the childbirth journey. The concept of silence linked all these themes. Women sometimes experienced re-enactment of abuse through intimate procedures but these were not necessarily problematic in themselves. How they were conducted was important. Women also experienced re-enactment of abuse through pain, loss of control, encounters with strangers and unexpected triggers…Maternity care was reminiscent of abuse for women irrespective of whether they had disclosed to midwives and was not necessarily prevented by sensitive care. Recommendations: As staff may not know of a woman’s history, they must be alert to unspoken messages and employ ‘universal precautions’ to mitigate hidden trauma.	Moderate
46a. Murphy 2014 [104] (46b. Steele 2016* [105], 46c. Steele 2010 [106] 46d. Murphy 2013 [107])	USA (Bronx, New York, ’clinical’ sample referred with child welfare concerns and a ’community’ sample)	75–118 mothers (clinical sample contains higher number of African American and Hispanic women who did not complete high school)	Unclear	Emotional abuse, physical abuse, sexual abuse, physical neglect, emotional neglect, household dysfunction (witnessing DV, divorce, mental illness, substance abuse, incarceration) (ACE, CTS, CTQ).	Cross-sectional and prospective study	**Assessment tool evaluation (1)** Examine relationship between ACE and AAI across both high and low risk populations (Murphy 2014) **(2)** consider the possible additive effect of ACEs, poverty, and clinical levels of parenting stress (Steele 2016).	**(1)** ACE responses were internally consistent. In the clinical sample, 84% reported ≥4 ACEs compared to 27% among the community sample. AAIs judged U/CC occurred in 76% of the clinical sample compared to 9% in the community sample. When ACEs were ≥4, 65% of AAIs were classified U/CC. Absence of emotional support in the ACEs questionnaire was associated with 72% of AAIs being classified U/CC. As the number of ACEs and the lack of emotional support increases so too does the probability of AAIs being classified as U/CC. **(2)** Parenting distress and ACEs were significantly higher in the low SES group; yet, even after controlling for SES, higher ACE scores explained variance in parental distress.	Low
47a. Muzik 2013 [108] (47b. Muzik 2013b* [109], 47c. Seng 2013* [110], 47d. Fava 2016* [14], 47e. Malone 2010* [111], 47f. Malone 1996 [112], 47g. Sexton 2015* [113], 47h Sexton 2017 [114], 47h Seng 2009 [115], 47i Malone 2015 [116], 47j. Swanson 2014* [117], 47k Hairston 2011* [118])	USA (Prenatal care clinics in mid-Michigan, 2005–2010)	52–566 women participating in Maternal Anxiety during the Childbearing Years study (MACY) longitudinal study and STACEY (PTSD) (>18 years, non-psychiatrically referred, predominantly caucasian, college-educated, married).	Pregnancy to 3 years postpartum	Emotional, physical or sexual abuse, or physical or emotional neglect <16 yrs (varying proportions affected dependant on analysis sample for each paper, but 65% total sample reporting abuse) (CTQ)	Mixed methods (Prospective cohort and Interviews)	**Parent suggestions about support after CT**: (**1)** To understand more about health care preferences of trauma-exposed women in the early postpartum period through qualitative interviews (Muzik 2013). **Investigate intergenerational pathways**: **(2)** to examine the trajectory of bonding impairment across the first 6 months postpartum in the contexts of maternal risk, including maternal history of childhood abuse and neglect and postpartum psychopathology, and to test the association between self-reported bonding impairment and observed positive parenting behaviours (Muzik 2013b) **(3)** to articulate the frequency and valence of new mothers’ general and parenting PTC, and examine whether maltreatment characteristics or concurrent demographic risk were related to PTC (Fava 2016). (4**)** examine the association between a mother’s history of CM (“ghosts” in her childhood) and her subsequent prenatal maternal representation of her child (i.e., the subjective way that a pregnant woman internally represents both who her child is and her relationship with the child and the impact on parenting (Malone 2010); **(5)** present the results of a prospective cohort study that quantified and modelled intergenerational patterns of trauma (Seng 2013); **(6)** associations between resilience, CM severity, mental health symptoms (postpartum PTSD and MDD), and positive functioning (self-perceptions of childrearing mastery and global family functioning), hypothesizing significant main effects for resilience and CM severity on outcomes; and whether resilience would moderate relationships between CM and maternal illness and health sequelae postpartum (Sexton 2015). **(7)** examine sleep complaints in postpartum women with a history of childhood trauma relative to postpartum women without a history of childhood trauma to determine whether sleep was differentially affected by the type of childhood trauma experienced, and to understand the contribution of PTSD to sleep complaints (Swanson 2014); and **(8)** investigate the role of infant sleep behaviours in IGTT, examining the hypothesis that infant sleep problems play a role in mother-infant relationship and other behavioural measures linked to the subsequent development of psychopathology (whether infants of mothers with a history of child abuse (with or without PTSD), are more likely to show sleep disturbances at 4 months, and whether sleep disruption at this age modulates the effects of mothers’ psychiatric symptoms on factors implicated in IGTT, such as bonding with the infant and child behavioural problems (Hairston 2011).	**(1)** Participants described ambivalence about seeking help but also a sincere desire for healing, coupled with hope for the future. This tension was apparent in the discussions highlighting the importance of access to experienced, non- judgmental, and knowledgeable health and social care staff and volunteers, the wish for both formal, integrated physical and mental health services, and for informal opportunities to meet other trauma-exposed mothers in a non-stigmatizing, child-friendly setting. Finally, positive relationship-building, respect, and safety were identified as key elements of services critical to counteract trauma-related shame and mistrust in others. **(2**) All women independent of risk status increased in bonding to their infant over the first 6 months postpartum; however, women with postpartum psychopathology (depression and PTSD) showed consistently greater bonding impairment scores at all times points. The 6 months assessment bonding impairment and observed parenting behaviours were significantly associated (Muzik 2013b) **(3)** General PTC were more likely to include negative and positive changes; parenting PTC were more likely to be exclusively positive. Indicators of more severe CM (parent perpetrator, more CM experiences) were related to parenting but not general PTC. Concurrent demographic risk moderated associations between number of CM experiences and positive parenting PTC such that among mothers with more CM experiences, demographic risk was associated with stronger positive parenting PTC (Fava 2016). (**4**) Controlling for domestic violence (DV), distorted prenatal representations were associated with higher rates of self-reported childhood physical neglect. DV moderated the relationship between representations and CM, such that women who were exposed to DV during pregnancy and had distorted prenatal representations were least likely to report childhood physical and sexual abuse (Malone 2010). (**5**) Posttraumatic stress in pregnancy, alone, or comorbid with depression, is associated with postpartum depression. Postpartum depression alone, or comorbid with posttraumatic stress, was associated with impaired bonding. In both models, higher quality of life ratings in pregnancy were associated with better outcomes, while reported dissociation in labor was a risk for worse outcomes. The effect of a history of childhood maltreatment on both postpartum mental health and bonding outcomes was mediated by pre-existing mental health status (Seng2013). (**6**) Resilience, childhood trauma severity, and their interaction predicted postpartum PTSD and MDD. In those with highest resilience, no mothers met criteria for postpartum MDD, irrespective of childhood trauma, while for those with lowest quartile of resilience, 25% with lowest CTQ severity and 68% of those with highest CTQ severity were depressed. The CD-RISC, but not the CTQ, was predictive of postpartum sense of competence. The CD-RISC and the CTQ were predictive of postpartum family functioning, though no moderating influence of resilience on childhood trauma was found. (Sexton 2015). **(7)** Participants who reported childhood neglect or physical abuse (regardless of sexual abuse) were significantly more likely to endorse difficulty falling asleep and staying asleep relative to participants who were not exposed to childhood trauma. Furthermore, PTSD was associated with sleep problems. (Swanston 2014). (**8**) Infants of PTSD+ mothers scored higher on the CSHQ and had more separation anxiety around bedtime than PTSD- and CON, and the severity of their symptoms was correlated with the degree of sleep disturbance. Maternal postpartum depression symptoms mediated impaired mother-infant bonding, while infant sleep disturbance contributed independently to impaired bonding. Poorer mother-infant bonding at 4 months predicted more behavioural problems at 18 months (Hairston 2011).	Moderate
48a. Nuttall 2015 [119] (48b. Nuttall 2012 [120])	USA (primary care facilities in South Bend, Indiana; Kansas City, Kansas; Kansas City, Missouri; Washington, DC; and Birmingham, Alabama, 2002–2007).	374 first time adolescent and adult mother-infant dyads (62% African-American and 16% Hispanic, only 56% completed high school, 59% sole parents)	Birth to 36 months postpartum (parenting assessment at 18 months)	Emotional, physical or sexual abuse, or physical or emotional neglect (CTQ).	Longitudinal/Prospective cohort	**Investigate intergenerational pathways**: whether the association between maternal parentification history and warm responsiveness is mediated by maternal knowledge of infant development in first time mothers.	Maternal retrospective reports of higher engagement in parent roles in family of origin were associated with poorer knowledge of infant development across the first 18 months of parenthood and, in turn, less warm responsiveness with 18-month-old children. However, maternal parentification history did not significantly influence changes in maternal warm responsiveness across the transition to parenthood, providing important empirical support for parentification theory and indicating the importance of maternal knowledge of infant development in the context of maternal parentification history.	Moderate
49a. Schechter 2008 [121] (49b. Schechter 2011*[122])	USA (hospital-based mental health clinic for very young children (aged 0–5 years) and their families, 2000–2001).	41 mother-infant dyads referred for parenting risk concerns with a parental history of maltreatment (88% Hispanic and 12% African-American, 54% did not complete high school, 68% received public assistance).	8 to 50 months postpartum	Physical and/or sexual abuse and/or domestic violence during childhood, and/or physical and/or sexual assault in adulthood (100%).	Cross-sectional study	**Investigate intergenerational pathways**: (**1**) To determine whether maternal violence-related posttraumatic stress disorder (PTSD), reflective functioning (RF), and/or quality of mental representations of her child predicts maternal behaviour within a referred sample of mothers exposed to interpersonal violence and their children (aged 8–50 months) (2008). (**2**) to understand if greater severity of maternal posttraumatic stress symptoms (PTSS), related to maternal report of interpersonal violence, mediates the effects of such violence on (a) child PTSS as well as on (b) child externalizing and internalizing symptoms (2011).	(**1**) Although maternal PTSD and RF impacted mental representations, no significant associations were found between PTSD, RF, and overall atypical caregiving behaviour. Severity of maternal PTSD was, however, positively correlated with avoidant caregiving behaviour. This suggests maternal mental representations of her child may be useful risk indicators that mark dysregulation of trauma-associated emotions in the caregiver (2008) (**2**) Paternal violence accounted for 15% of the variance of child PTSS. However when maternal PTSS is included in the multiple regression model, father’s being violent becomes less significant, while maternal PTSS remains strongly predictive (2011).	Moderate
50a.Smith 2016 [123] (50b. Yonkers 2012 [124])	USA (137 obstetrical practices and hospital-based clinics throughout Connecticut and Western Massachusetts, 2005–2009)	2303 women (predominantly caucasian (76%), married (90%) and college-educated (mean 15 years)).	Pregnancy to 8 weeks postpartum	Fifty percent of women reported experiencing at least one adverse childhood event prior to the age of 18: 21% (n = 493) reported one, 11% reported two and 17% of women (n = 397) reported exposure to three or more adverse childhood events prior to the age of 18 (Table 1). The most common reported event was substance abuse by a parent (22%), followed by sexual molestation/abuse (16%).	Prospective cohort	**Investigate intergenerational pathways**: association between adverse childhood experiences (ACEs) and pregnancy outcomes; and to explore mediators of this association including psychiatric illness and health habits.	Each additional ACE decreased birth weight by 16.33 g and decreased gestational age by 0.063. Smoking was the strongest mediator of the effect on gestational age. This supports two hypotheses (1) ACEs themselves may result in adverse birth outcomes; (2) the health behaviours of people who have experienced ACEs, are implicated in poor pregnancy outcomes and/or (3) ACEs result for some in adverse birth outcomes mediated by psychiatric disorders and prenatal smoking.	Moderate
51. Thomas 2012 [125]	USA	6 child maltreatment survivors (4 caucasian and 2 Hispanic, all completed high school and currently employed or studying a higher degree, 5 married)	Unclear	Multiple types of abuse (100%).	Qualitative (Interviews)	**To examine the phenomenon of anger** and its role in the recovery process of 6 midlife women.	A typology was constructed, depicting 5 types of anger ((1) self-castigating anger; (2) displaced anger; (3) the anger of indignation; (4) self-protective anger; and (5) righteous anger). Anger ranged from non-productive, self-castigating behaviour to empowering, righteous anger that enabled women to protect themselves from further abuse and to advocate for abused children. Anger intensity waxed and waned across the years of the healing trajectory, peaking for some women in their 20s and 30s.	Moderate
52. Thornberry 2013 [126]	USA (Rochester, NY, 1988 ongoing)	401 parents aged 21–23 participating in longitudinal youth study (proportionately stratifying on residence in high crime areas of the city, 70% African-American and 15% Hispanic, 59% living in poverty).	Unclear	Maltreatment based on official records (20.8%).	Longitudinal/prospective cohort	**Investigate intergenerational pathways**: (1) degree of intergenerational continuity in maltreatment; (2) if SSNRs in early adulthood decrease the likelihood of perpetration among maltreated individuals (i.e., SSNRs are direct protective factors); and (3) if SSNRs in early adulthood offset or buffer the negative effect of maltreatment on perpetration (i.e., SSNRs are buffering protective factors).	There is a significant relationship between maltreatment victimization and maltreatment perpetration. Three of the five SSNRs investigated (relationship satisfaction, parental satisfaction, and attachment to child) served as direct protective factors, significantly reducing risk for those who had been maltreated. However, SSNRs did not serve as buffering protective factors.	Moderate
53. Widom 2015 [127]	USA (Midwestern county, 1967–1995)	1196 parents recorded as maltreatment victims in CP records and a matched comparison group from the same neighbourhoods.	Unclear	Child protection substantiation	Prospective cohort	**Investigate intergenerational pathways**: To assess identification of maltreatment based on child protective service (CPS) agency records and reports by parents, nonparents, and offspring.	The extent of the intergenerational transmission of abuse and neglect depended in large part on the source of the information used. Individuals with histories of childhood abuse and neglect have higher rates of being reported to CPS for child maltreatment but do not self-report more physical and sexual abuse than matched comparisons. Offspring of parents with histories of childhood abuse and neglect are more likely to report sexual abuse and neglect and that CPS was concerned about them at some point in their lives.	High
54. Wilkes 2002 [128]	USA (Arizona)	20 parents (only 2 male) reporting child maltreatment in response to community advertisements (Predominantly caucasian (85%), married (65%), and completed high school (90%).	Unclear	Most often both verbal and physical (75%).	Cross-sectional study	**Investigate intergenerational pathways**: The nature of change operating in this particular case of resilience, moving from abused child to non-abusive parent; factors that have influenced the ability to change and disrupt the pattern of abuse; and family influences, personality traits, and positive and/or negative coping strategies that may have helped or hindered individuals in the change process.	The highest rates of ’negative’ coping behaviours reported were overeating (65%), smoking (50%), compulsive work (50%), and oversleeping (40%). Participants also reported engaging in positive coping behaviours such as counselling/therapy (65%), volunteering (65%), spiritual or religious activities (55%), meditation (50%), and exercise (50%). In the “other” category of positive coping behaviours, several participants listed reading and learning about abuse.	Very low
55a. Zuravin 1996 [129] (55b. Zuravin 1987 [130], 55c. Banyard 1999* [131], 55d. Banyard 2003* [132])	USA (1970 onwards)	152–237 mothers receiving financial aid with CPS involvement and matched comparisons (predominantly African American (>74%) and sole parents (>60%), high proportion did not complete high school (40%))	Children <12 years	Neglect, physical abuse and sexual abuse (CPS substantiation) (50% with ’matched controls').	prospective cohort	**Investigate intergenerational pathways**: (**1**) dose relationship between attachment and continuity (Zuravin 1996). (**2**) whether adult risk variables and negative childhood family relationships and childhood victimization affect women’s depression and self-esteem; and, for survivors of maltreatment, whether positive coping resources such as religion, social support and a less external locus of control predict lower depression and less problems with self-esteem (Banyard 1999). (**3**) the impact of cumulative adversity, depression and adult trauma on parenting outcomes; and protective factors (Banyard 2003).	(**1**) Poorer quality attachment in childhood predicted transmission of physical abuse only. There was no interaction effect for attachment quality with any of the other four definitions of maltreatment. Given equally high levels of attachment, mothers who were sexually abused or neglected were about 20% less likely to perpetuate the cycle than mothers who reported no or infrequent severe beatings (Zuravin 1996). (**2**) PCT predicated higher depression, lower social support & self-esteem. Social support and a less external locus of control were protective in function (Banyard 1999). (**3**) Higher rates of trauma exposure were related to decreased parenting satisfaction, reports of child neglect, use of physical punishment, and a history of protective service reports. These links were partially mediated by the relationship between trauma exposure and increased maternal depression. Social support and taking care of one’s own needs was protective against for parental satisfaction, while maternal depression was a mediator (Banyard 2003).	Low
56a Ammerman 2016 [133] (56b Ammerman 2012a [134], 56c Ammerman 2012b [135])	USA (Southwest Ohio and Northern Kentucky)	93 mothers	2–10 months postpartum	Emotional, sexual and physical abuse, emotional and physical neglect.	Cross sectional study within an RCT	**Investigate intergenerational pathways:** to examine the moderating effects of child maltreatment history on depression, social functioning, and parenting in mothers participating in a clinical trial on home visiting (2016); and contrast depressed and non-depressed mothers enrolled HV program on parenting stress, quality of home environment, social network, and psychiatric symptoms (2012b).	2016: A moderating effect of maltreatment on treatment outcomes was found for physical abuse and parenting and emotional abuse and social network size. 2012b: Path analyses revealed a path linking childhood trauma, depression, and parenting stress, which was mediated by social network.	High
57a Noll 2007 [136] (57b Kwako 2010) [137]	USA (Washington DC)	124 mothers	5 months to 10 years postpartum.	Child sexual assault verified by medical or police authorities.	Longitudinal study of CSA survivors.	**Investigate intergenerational pathways:** To compare preterm birth rates across offspring born to mothers who were sexually abused in childhood (Noll 2007); and to explore link between CSA, maternal attachment, and infant attachment (Kwako 2010).	Preterm delivery rates were higher for the CSA group (Odds 2.80 CI 1.44, p<0.05) (Noll 2007). Children of CSA survivors were more likely to have extreme strategies of attachment than the children of non-abused mothers (Kwako 2010).	High

## Results

**Fig 1:**
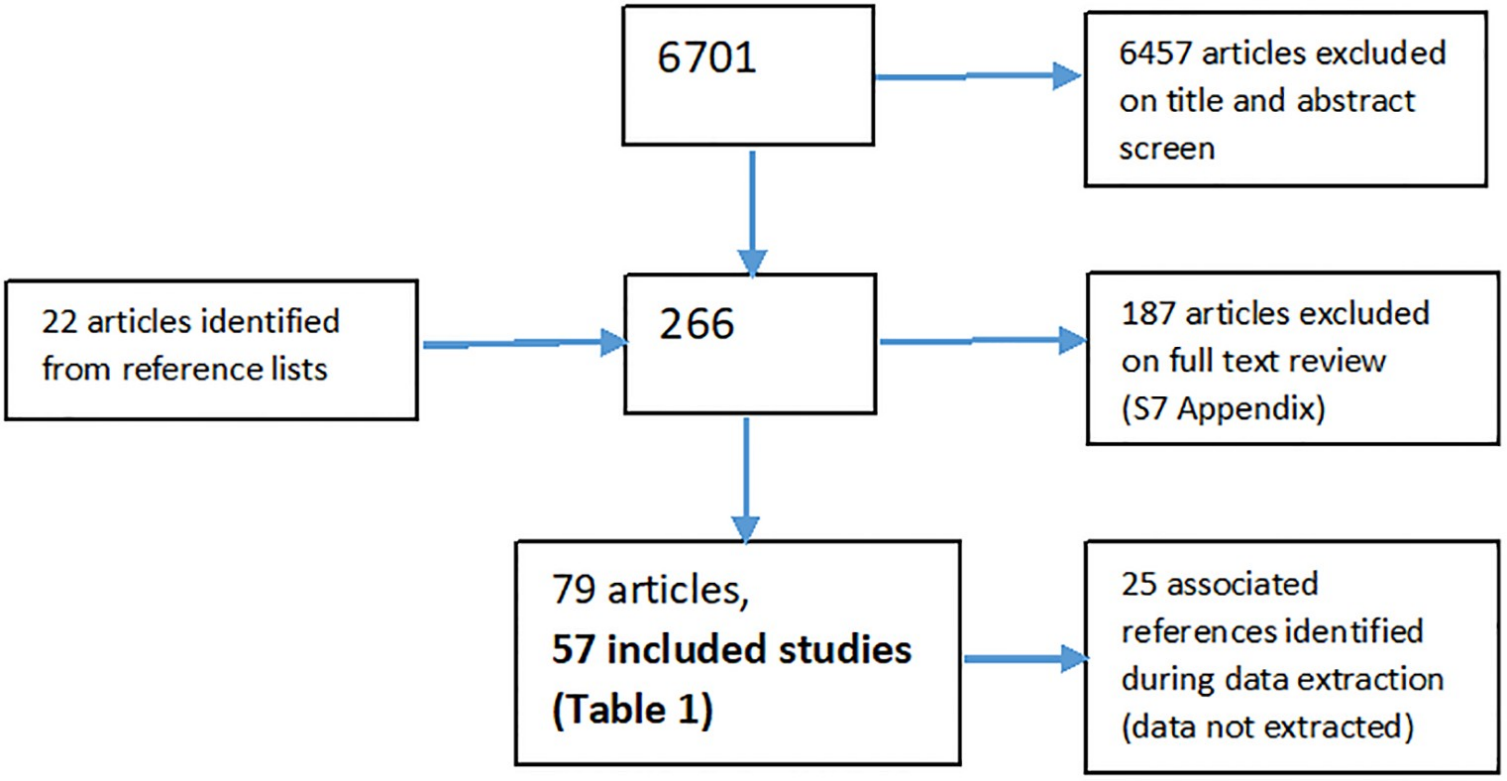
Flow chart of included studies.

Fig 1 shows the flow of studies at each stage of the review. From 266 full text articles, 57 studies (79 articles) were included. Reasons for exclusion of the remaining 187 are listed in S7 Appendix.

### Description of included studies

**Settings:** Over 20 000 parents/parent-child dyads participated in the 57 included studies. The majority of studies were conducted in the United States (n = 44), with other studies in Australia (n = 3), Canada (n = 3), Germany (n = 1), Netherlands (n = 2) and the United Kingdom (n = 4). Most studies have been published in the most recent decade (2010+) (n = 27), with fewer studies published in earlier decades; 2000–2009 (n = 17), 1990–1999 (n = 11) and before 1990 (n = 2).

Parents were recruited from a wide range of settings, including; prenatal/maternity clinics (n = 12), paediatric/special care nurseries (n = 2), community health and primary care clinics (n = 5), general communities (n = 7), Indigenous communities (n = 1), mental health programs (n = 5), child welfare agencies (n = 5), parenting programs (n = 4), financial assistance programs (n = 3), youth foster programs (n = 2), schools (n = 2), relationship support centre (n = 1), parents self-help group (n = 1), correctional centre (n = 1), health visitor program (n = 1) and an addiction treatment centre (n = 1). The setting was unclear in four studies.

**Participants:** Forty-five of the 57 included studies comprised only mothers and ten studies included both parents. In the remaining two studies it was unclear whether the participants were mothers and/or fathers. No studies included only fathers, extended family, partners of same-sex couples or other carers. Forty-eight studies reported sociodemographic characteristics of the participants. The mean age of parents was 28 years, with an age range of 12–62 years. Forty-three studies reported parent *ethnicity*; 23 studies reported a majority (>50%) of Caucasian parents and 20 studies a majority of African-American and/or Latino parents. Indigenous parents were identified in six studies (<7%), and included Native American, First Nation Canadian and Aboriginal and Torres Strait Islander Australian parents.

*Parent education level* was reported in 30/57 studies. In eight studies the majority (>50%) of parents were college graduates, in 12 studies the majority were high school graduates, and in 10 studies the majority reported not completing high school. In 14 of these 30 studies there was a relatively high proportion of parents (>20%) who had not completed high school. A measure of *socio-economic status (SES)* of parents was reported in 35 studies, and in 22 studies the majority of parents reported experiencing socio-economic disadvantage. The *marital/relationship status* of parents was reported in 34 studies. The majority (>50%) were classified as single/sole parents in 14 studies; with a further three studies reporting more than 20% parents as single/sole parents.

Thirty-eight studies identified parents during the perinatal period (pregnancy to two years postpartum). In 19 studies the age of the children was unclear. No studies explicitly included parents before pregnancy. Twenty-three studies involved parents during pregnancy and up to six weeks postpartum, 30 studies involved parents from 6 weeks postpartum to 12 months after birth, and 23 studies involved parents from 13 to 24 months postpartum.

Thirty-five studies included parents both with and without a history of childhood maltreatment, 20 exclusively included parents with histories of childhood maltreatment, and two were unclear (reports of resolved and unresolved trauma and childhood maltreatment as one of several risk factors required for inclusion). Childhood maltreatment experiences reported by parents included; emotional abuse (17 studies), sexual abuse (39 studies), physical abuse (44 studies), neglect (emotional (9 studies), physical (9 studies) or otherwise unspecified (14 studies)), exposure to domestic/intimate partner violence (IPV) (14 studies) or witnessing other violence (2 studies) and ‘Adverse Childhood Experiences (ACEs)’ (5 studies). Experiences and definitions of childhood maltreatment within included studies also incorporated; ‘harsh parenting’ (2 studies), parental suicide and ‘bizarre punishments’ (1 study), household dysfunction (1 study), having a guardian in trouble with the law or in jail or homeless (1 study), living in poverty or apart from parents before 16 years of age (1 study), unresolved trauma (1 study), and adult sexual and/or physical assault (2 studies) or ‘adult single event trauma’ (1 study). Five studies relied on retrospective reports of child protection substantiations, and one study exclusively included parents who had themselves been removed from their families of origin during childhood.

**Types of studies:** The majority of studies used a quantitative descriptive design (36/57 studies). Of the remaining studies, 16 were qualitative, three were RCTs of interventions (and one associated article), and two evaluated measurement/assessment tools (one associated article).

**Outcomes reported by included studies:** Most outcomes were measured at the level of individuals (or parent-infant dyads) (52 studies), one study collected outcomes at a family level and in two studies this was unclear. Only five studies reported outcomes separately according to socio-demographic factors (based on PROGRESS-plus equity criteria), including maternal age (4 studies), ethnicity (4 studies), educational level (3 studies), relationship status (4 studies), socioeconomic status (2 studies). However many studies conducted analyses in which the associations with outcomes that were estimated were adjusted for socio-demographic factors. See Table 1.

A summary of evidence under each of the main outcomes (theories, risk and protective factors, parents’ views, interventions and assessment tools) are outlined briefly below to provide an ‘evidence map’. Evidence will the synthesized in detail using methods appropriate to each study type in subsequent comprehensive systematic reviews (S1 Appendix).

### 1. Theoretical frameworks

Sixteen different theoretical frameworks were identified across 14 studies in this review. A brief description of the main theories are listed in Table 2 and outlined narratively below.

**Table 2:** Theoretical constructs identified in included studies: An evidence map.

Theoretical construct (reference)	Characteristics of included study table (Table 1) study ID
Attachment theory (Bowlby, 1969)	6, 9, 25a, 25b, 34, 40a, 41a, 55a, 55d
Belsky’s sociological model (Belsky, 1978)	8a, 9, 55c
Ghosts in the nursery (Fraiberg et al., 1975) and Angels in the nursery (Alicia F. Lieberman et al., 2005)	2, 39, 47i
Relational development theories (J. D. Bartlett & Easterbrooks, 2015)	8a
Object relations theory (Westen, 1991)	9
Resilience theories and frameworks	8a, 16, 44
Social learning theory (Albert Bandura & Huston, 1961)	9, 16, 25a, 25b
The family systems model	33, 47a
Child Sexual Assault (CSA) Theoretical Healing Model	24, 47d
Anger theories	51

***Attachment theory*** [138] was the most common theory identified among studies in this review [45, 129, 132]. Attachment theory was used to try to understand effects of adversity and protective factors on continuation or discontinuation of intergenerational cycles of maltreatment [41, 72, 73, 132]. Iyengar et al. [86] examined the role of *attachment reorganisation*, where parents were actively changing their understanding of past and present experiences and moving toward ‘earned attachment security’ and ‘resolved trauma’ as assessed on the Adult Attachment Interview (AAI) and they found all mothers who were ‘reorganising’ had infants with secure attachment. Lyons-Ruth and Block [94] found infants with insecure attachment born to mothers with a history of childhood maltreatment displayed predominantly disorganised attachment, while infants with insecure attachment born to mothers without a history of childhood maltreatment displayed predominantly avoidant strategies. Madigan et al. [96] examined the stability of disorganised attachment in a sample of adolescent mothers transitioning to parenthood and concluded adolescents seemed to find it harder to re-organise attachment and ‘resolve trauma’ than older mothers.

***Belsky’s sociological model*** [139] was used in several studies as a theoretical framework to investigate the dynamic nature of factors affecting discontinuity and continuity of childhood maltreatment [43, 45, 132].

A ***ghosts in the nursery*** [140] metaphor has been used to describe the way parents re-enact their childhood histories from unremembered early relational experiences [37].

Malone et al. [111] suggested that relational theories provide support for how the “ghosts” of one’s past develop and may become a persistent presence in future relationships. They suggest that a safe supportive relationship or “holding environment” will be needed before parents can begin to reflect on how abuse in their own childhood influences their lives now, and argues interventions that help parents recognize and resolve the negative experiences from their past will enable the formation of a positive relationship with their child [111]. These schemas, or internal working models, initially develop through interpersonal interactions with primary caregivers, including experiences of loss and abuse [138], and are then kept outside of consciousness by defensive strategies that protect an individual.

An ***Angels in the nursery*** [93] metaphor was proposed to reframe ‘ghosts in the nursery’ to also consider positive factors. Leiberman et al. described the ways in which early benevolent experiences with caregivers can work as protective forces even in the face of overwhelming trauma, and how examining these consciously can be used as a powerful healing tool, by placing the traumatic cues within the larger perspective of nurturing and growth-promoting experiences [93].

***Relational development theories*** were used to consider multiple aspects of a developmental system (e.g., individual history, social relationships, and environmental context), as well as the parents current social context. These may be particularly important given that parents with a history of childhood maltreatment may draw on fewer support networks and experience higher levels of parenting stress [43].

***Object relations theory*** was used to help understand aspects of psychological functioning that support adaptive relational development [45]. Baumgardner suggests that this is, in essence, what Bowlby [138] referred to as an ‘internal working model’, a cognitive framework comprising mental representations for understanding the world, self, and others [45].

***Resilience theories and frameworks*** [141] were used as a way of understanding protective factors associated with discontinuities in the intergenerational transmission of trauma [43, 57, 101]. Authors argued that understanding risk factors is only half of the picture, and understanding factors associated with resilience and exceptions is critical for designing effective interventions and policies [43, 57]. Bysom suggested these can be viewed as existing on a continuum, with variability exhibited throughout a lifetime [57].

***Social learning theory*** [142] was used to explain how some parents replicate the behaviour of their own parents [45, 57, 72, 73]. However, Bysom [57] argued it was limited in its ability to explain the exceptions and discrepancies seen by most parents with a history if child maltreatment and suggested ***social cognitive theory*** [143] may be more helpful to distinguish and explain some of these exceptions, but is inadequate for explaining resilience.

***The family systems model*** [85] was used in one study [85, 108] to help explain differences between maltreating and non-maltreating families in a special care nursery setting, with ‘differentiation of self’ identified as an important factor in discontinuing the cycle of violence.

A ***Child Sexual Assault (CSA) Theoretical Healing Model*** was constructed from qualitative research [71]. In developing the model, the authors also drew on literature about *post-traumatic growth*, arguing that survivors not only cope but engage in dynamic processes that include growth and recovery. Their model incorporates constructs from positive psychology, including perceived self-efficacy, personal control, altruism and empathy, and spirituality. Fava et al. [14] explored themes of *post-traumatic change* and found that parents’ reports of post-traumatic changes during the postpartum period (four to 18 months) were more likely to be exclusively positive, compared to other periods of the life-course when post-traumatic changes were likely to include both negative and positive changes.

***Anger theories*** were explored in a qualitative study to help understand the role of anger in the healing trajectory from childhood maltreatment [125]. The authors constructed a typology depicting five types of anger ranging from non-productive self-castigating and displaced behaviour to empowering indignant, self-protective and righteous anger that enabled women to protect themselves from further abuse and to advocate for abused children [125].

### 2. Life-course and intergenerational pathways (risk and protective factors)

**Fig 2:**
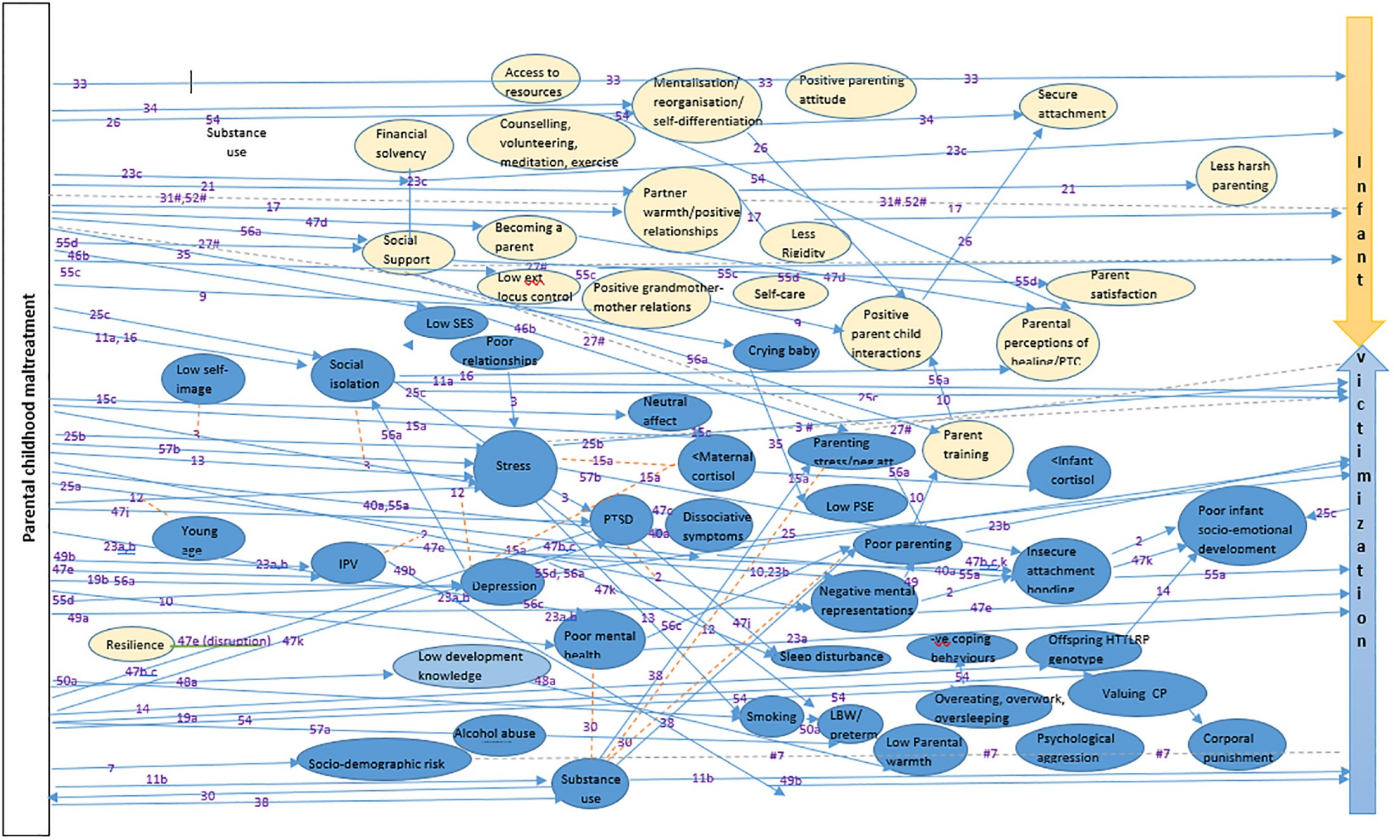
Summary of risk and protective factors that mediate/moderate life-course and intergenerational pathways following parental history of childhood maltreatment.

Thirty-eight studies described risk and protective factors that mediate and/or moderate pathways from a parental history of childhood maltreatment to parental and infant outcomes in the perinatal period. These are briefly outlined below and depicted in Fig 2 which includes the study ID numbers for the COIS Table 1.

**Distal risk factors** included ***socio-demographic deprivation*** [42, 105], ***young parental age*** [68, 69], ***social isolation or poor social functioning*** [49, 57, 74, 133, 135], ***substance use*** [50, 82, 92], ***alcohol use*** [136], ***smoking*** [52, 123, 128], ***intimate partner violence*** [61, 68, 69, 111, 122], ***poor relationships*** [38], ***a lack of child development knowledg****e* [119], ***poor mental health*** [46, 68, 69, 135], ***stress*** [9, 38, 52, 73, 74, 137], ***depression/neutral affect*** [56, 68, 69, 109, 110, 132, 133, 135], ***and PTSD or dissociation symptoms*** [38, 54, 94, 109, 110, 117, 129].

These were associated with **proximal risk factors** such as ***decreased parenting self-efficacy in response to infant crying*** [87], ***parental sleep problems*** [117, 118], ***parenting stress*** [92, 105, 135], ***negative mental representations of the infant*** [37, 46, 69], ***poor parenting practices*** [69, 92, 121, 133], ***low parental warmth*** [50, 119, 120], ***negative coping behaviours such as oversleeping*, *overeating and overworking*** [128] and ***valuing corporal punishment*** [60].

These factors were associated with; ***offspring genotype*** [53] ***and cortisol changes*** [54], ***low birth weight and preterm birth*** [9, 123, 136], ***insecure attachment and bonding*** [37, 94, 109, 110, 118, 129, 133, 137], ***infant victimization*** [49, 50, 68, 69, 73, 74, 111, 122, 129, 132], ***and poor infant socio-emotional development*** [37, 53, 74, 118].

There were a range of **distal protective factors** that mediate and/or moderate pathways to perinatal outcomes following a parental history of childhood maltreatment. These included; ***resilience*** [111], ***financial solvency*** [70], ***access to resources*** [85], ***social and family support*** [45, 70, 131–133], ***partner warmth and positive relationships*** [58, 66], ***a ‘low external locus of control’*** [131], ***less rigidity*** [58], ***practising self-care*** [132], ***attending counselling*, *meditation*, *volunteering or exercise*** [75, 128], ***mentalization*, *attachment reorganisation or self-differentiation*** [40, 75, 85, 86, 128] ***and parent training*** [46, 133].

These were associated with **proximal protective factors** including; ***more positive parent-child interactions*** [45, 46, 57, 75], ***parent satisfaction*** [132], ***a positive parenting attitude*** [85], ***parental perceptions of healing*** [14, 57, 128], ***secure attachment [75, 86, 133]***, ***less ‘harsh parenting’*** [66], ***and lower rates of infant maltreatment*** [58, 70, 85, 132]. Several studies examined but reported null findings for social support [76] and supportive relationships [83, 126] as protective factors.

### 3. Perinatal experiences described by parents with a history of childhood maltreatment and strategies used during the perinatal period to heal from childhood maltreatment and/or prevent intergenerational transmission of trauma

Eleven studies described perinatal experiences and/or strategies of parents with a history of childhood maltreatment. One study described maternal healing after CSA [71] and six studies reporting parents’ perceptions of factors that influence healing and/or ability to ‘break the cycle’ of intergenerational trauma [39, 57, 67, 71, 98, 101, 128]. Two studies reported women’s experiences of perinatal services [84, 102, 103] and one reported experiences of breastfeeding [65], specifically among women who had experienced CSA. A brief outline of the main themes are summarised and mapped to the COIS Table 1 study number in Table 3 below.

**Table 3:** Parents experiences in the perinatal period: Evidence map of qualitative studies.

	COIS study ID (Table 1)	
	Parent descriptions about ’breaking the cycle’ of childhood trauma	Parents descriptions of perinatal care after Childhood Sexual Assault (CSA)
**Experiences parents described regarding effects of childhood trauma in the perinatal period**		
Post-traumatic growth	24	
Parenting a healing opportunity/living family legacy	24	
Healing process is complex, including parenting transition	4,24	
Birth and parenting journey difficult for most survivors of CSA		4
Disclosure not always beneficial/stigma	22,24	
Fear of child protection agencies	4	
Feeling of lack of parenting knowledge/skills	44	
Negative sense of self-worth (including guilt/shame)	4,44	
Lack of control	44	
Lack of trust leading to friendship/therapeutic relationship challenges	22,44	4,32
Difficulty negotiating boundaries, including lack of caring for oneself and taking responsibility for others behaviour (including children)	44	
Loss and abandonment feelings	44	32
Many aspects of care re-traumatising		32,45a
Disbelief/betrayal of confidence/invalidation of experiences		32
Experiencing punitive responses to self-harm from care-providers		32
Unexpected triggers from intimate procedures, pain, encounters with strangers, lack of control, breastfeeding		45a, 20
Breastfeeding important to help improve maternal-infant attachment after CSA (but can trigger trauma responses)		20
Ability to breastfeed validates body as ’good or bad'		20
System-level barriers to care (lack of services, poor access)		32
**Strategies parents described using to heal and/or break cycles of trauma**		
Understanding, intellectual competence, meaning making and storying, including determining sexuality	22,24,44,54	
Using conscious strategies to tackle effects (e.g. leaving violent partners)	24,32	
Determination to parent differently (including considering safety and sensitive communication)	4,22,32,42a,44	
Spirituality/religion	16,24,54	
Helping others/volunteering/advocacy	24,51,54	
Reducing isolation and increasing support (relationships and social)	4,16, 22, 32 44	
Self-care/time out/meditation/reading/exercise	4,16,54	
Psychotherapies (mixed responses about value)	4,54	
Speaking up to empower self	22	
Critical parenting experiences coupled with access to resources ’at the right time'	44	
Overeating, compulsive work, oversleeping	54	
Smoking	54	
Anger	51	

#### Parents’ experiences

Parents described experiences of ***post-traumatic growth*** [71] and the unique ***opportunities for healing*** through parenting for adolescents transitioning out of foster care [39]. Parents’ also described ***challenges related to disclosure of child sexual assault*** [71] and child maltreatment, such as; ***stigma***, awareness of the time pressures on staff in maternity services [67], ***fear of child protection agencies [39]***, ***lack of parenting knowledge and skills*, *and mental and physical health problems*** [101]. Parents’ described ***difficulties negotiating boundaries*** which was perceived as contributing to being unable to pay attention to self-care, and taking responsibility for the behaviour of others, particularly their children [101].

Parents described the impact of having a ***negative sense of self-worth*** on parenting [101] which included ***feelings of guilt and shame*** that were shaped by previous experiences [39]. Experiencing a ***lack of control*** within the context of parenting was identified as one of the most re-traumatising aspects of perinatal care and breastfeeding [65, 103]. Gaining a sense of control was seen as critical to overcoming these types of stressors [65, 103]. CSA survivors described how ***breastfeeding could validate their views of their bodies as ‘good’ or ‘bad***’ depending on the ‘success’ of their breastfeeding [65].

***Lack of trust in relationships*** was a common issue that impacted parents’ ability to make friends which frequently led to feelings of isolation [67]. These difficulties in trusting others were also seen to impact on developing therapeutic relationships with perinatal care providers [39], often exacerbated by fears of child protection agencies [84]. Parents also described challenges related to dealing with feelings of loss and abandonment [101] which also impacted on experiences of maternity care [84].

Parents described how some experiences could be re-traumatising [84, 103]. These included encountering ***responses of disbelief and invalidation of their past experiences***, ***punitive responses to self-harming and betrayal of confidenc***e [84]. Women described a range of ***unexpected triggering of trauma responses*** in birth and parenting, including intimate procedures, encounters with strangers, pelvic pain [103], and distress at being unable to soothe their crying baby [84].

A range of ***system level barriers*** were also identified, including; lack of services (particularly psychotherapy), access problems (lack of transport and childcare), infrequent sessions with limited opening hours, short term contracts for staff, use of waiting lists and long waiting times in clinics [84]. There were mixed reports about the value of psychological therapies [39, 128] and concerns about the lack of alternative approaches to therapy (such as art therapy). Stigma and the chaotic lives of some parents were identified as individual level barriers [39]. Monaghon-Blout [101] suggested that critical parenting experiences often increased motivation for change, and if coupled with access to resources ‘at the right time’ could help to support positive healing.

#### Strategies parents describe using to heal or break the cycle of trauma

***Understanding the experience of trauma*, *‘storying’ and ‘meaning making’*** were common strategies used by parents to heal and prevent intergenerational transmission of trauma [67, 71, 128], conceptualised by Draucker et al. [71] as a specific healing phase. Other phases involved ***enacting conscious strategies to tackle the effects of trauma*** [71], such as leaving violent partners to ensure child safety [84] and promoting positive communication [67]. Draucker et al. [71] described a final healing phase where parents make a ***commitment to not pass on the effects of trauma*** to their children. Parents who had experienced maltreatment themselves consistently reported a strong desire and ***determination to parent differently*** with their own children [39, 67, 71, 84, 98, 101, 103]. However, some parents continued to disclose behaviours that were harmful, despite expressing these intentions [57, 98].

***Reducing isolation and increasing social support*** was also identified as critical to healing [67]. Parents reported that an important aspect of social support was increasing awareness of alternative methods of parenting, and that non-judgemental social support was the most helpful [57]. Parents also highlighted the ways in which issues such as lack of trust and a negative sense of self-worth could contribute to difficulties in establishing and maintaining friendships [101]. An important source of support for parents who had challenging relationships with intimate partners or their family of origin was having a ‘family of friends’. Also important were supportive interpersonal relationships [67, 101] and mentors [39].

***Spirituality*, *self-care and ‘centering on self’*** were identified as key aspects of healing [57, 71, 128]. Self-care activities included taking ‘time out’ [39], meditation, exercise and reading [128]. Parents also described ***helping others and volunteering*** as having a healing effect [71, 128].

Thomas et al. [125] explored the role of ***anger*** in healing, and suggested that ‘righteous anger’ could assist healing as it often motivated people to help others. ‘Self-protective’ and ‘indignant’ anger were seen as positive, while self-castigating and displaced anger were considered harmful [125]. There was consensus that ‘hanging on’ to any type of anger was harmful [125]. Parents also reported that speaking up about trauma could add to self-empowerment [67].

### 4. Perinatal interventions to support parents with a history of childhood maltreatment

Eleven studies provided evaluations or descriptions of strategies/interventions to support parents with a history of maltreatment in their own childhood. The main outcomes from these studies are briefly described below and summarised in Table 4. Details about the setting, population and overall confidence in the study findings are summarised in COIS Table 1. A comprehensive systematic review of intervention studies is planned and will include detailed synthesis of study outcomes and assessment of heterogeneity, barriers and facilitators (S1 Appendix). No economic evaluations were found.

**Table 4:** Perinatal interventions to support parents with a history or child maltreatment: Evidence map.

	Nurse home visiting	Parenting programs (e.g. Incredible Years; Head Start; Safe Mothers, Safe Children)	Cognitive Behavioural Therapy (CBT) for smoking cessation	Brief interpersonal therapy (IPT-B)	Psycho-therapy (general)	Parents’ self-help groups	Perinatal Support’ otherwise unspecified
Parental wellbeing measures*							
Postpartum depression*	5, 56a	18	13b	29a			
Anxiety/parenting stress*	5, 56c			29a			
Trauma symptoms		18					
Satisfaction and experiences of program^						44	
Participation in program		10					
Parenting self-efficacy/confidence^							
Substance use (including smoking)^		10	13b				
Breastfeeding	5						
Sudden Infant Death Syndrome (SIDs) knowledge	5						
Psychosocial/social function	56a,c		29a		37		
Interpersonal problems			29a				
Parent perceptions of what helped or hindered healing						44	
Parent-child interactions*	5	10			37		
Parenting skills	56a,c	10					
Secure attachment*					37		
Couple relationship/adjustment^							
Intimate partner violence (IPV)^							
Child wellbeing measures*							
Child maltreatment/reports to child protection^							
Hospitalisations^/use of health services							
Fetal Alcohol Syndrome^							
School achievement measures^							
**Other**							
Adverse outcomes/unintended consequences^							
Community violence^							
Cost, cost effectiveness^							
Child maltreatment disclosure							1
Child maltreatment assessment feasibility (provider views)		18					
Expression of interest in support/support preferences							1,47a
Home environment	5						
Therapist reflections					6,43		

Numbers correspond to study ID in COIS Table 1

*Primary outcomes from protocol

˄Secondary outcome from review protocol

One study analysed cross-sectional data from a parenting program intervention and found high rates of trauma exposure among the participating parents. Service providers found the associated screening process both feasible and beneficial Three formative studies asked parents whether they would like support and/or what type of support would be helpful. Adams [36] mail-surveyed 89 first-time mothers in the US, and found that of 15 percent who disclosed a history of childhood maltreatment, two thirds expressed interest in receiving support. Muzik et al. [108] undertook qualitative interviews with 52 trauma-exposed mothers and found that although women were often ambivalent and anxious about receiving formal help, they consistently expressed a desire for healing and hope. Women in the study identified a need for experienced, non-judgmental and knowledgeable support, which includes formal integrated mental and physical health services, and opportunities to meet other parents informally in a non-stigmatising child-friendly environment. Positive relationship building, respect and safety were described as key elements of support to counteract trauma-related effects of shame and mistrust [108]. In-depth interviews with seven members of a ‘parents anonymous’ self-help group [101] were analysed to develop a recommendations for ‘building a helping alliance’ within parent self-help groups. These included helping parents to understand how childhood maltreatment can interfere with establishing positive relationships, and enabling access to ‘the right support’ during critical periods [101].

The effects of five perinatal interventions were evaluated in RCTs. However, none of these parenting interventions were specifically designed for parents with a history of childhood maltreatment and the results reported here represent findings from subgroup analyses only. Armstrong et al. [40] evaluated an Australian ***nurse home-visiting*** intervention compared to standard community child health services (from 6 weeks postpartum) with 181 ‘vulnerable parents’ that included 33% mothers and 17% fathers with a history of childhood maltreatment. No differences were reported between groups for breast feeding initiation or duration, knowledge of SIDS, or use of health services. Those receiving nurse home visits were reported as having improved scores for depression, parenting stress, parent-infant interactions and home environment compared to the comparison group [40]. Ammerman et al [133] examined the moderating effects of child maltreatment history on depression, social functioning, and parenting in mothers participating in a home-visiting trial for mothers with depression in the United States. They found a number of main effects in which experiences of different types of trauma were associated with poorer parent functioning regardless of whether they were receiving the home-visiting intervention, suggesting a need for targeted strategies to support parents who have experienced child maltreatment. A study evaluating the effects of ‘the ‘*I****ncredible Years Parenting program***’ compared to the regular ‘Head Start’ curriculum (Puget Sound, US) with 421 parents, reported lower baseline parenting skills among parents with a history of childhood maltreatment, and presented cross-sectional data suggesting that this history did not impact on *participation rates* in the program [46]. Parents’ receiving the intervention demonstrated significant improvements in parenting behaviours compared to those receiving the standard ‘Head Start’ curriculum [46]. An RCT evaluating the effects of ***cognitive behavioural therapy (CBT)*** aiming to support smoking cessation in pregnancy reported improvements in postnatal depression scores, but no differences in smoking cessation for women receiving CBT compared to standard health education [52]. However, subgroup analyses suggests women with a history of childhood maltreatment were more likely to experience improvements in depression and less likely to stop smoking than women without [52]. Finally, an RCT of ***brief interpersonal therapy (IPT-B)*** for parents with a history of childhood maltreatment reported improvements in postnatal depression, anxiety, social dysfunction, interpersonal problems and insecure attachment among parents receiving the intervention, compared to parents allocated to the control group who received ‘usual care’ [20].

Three articles described case studies of individual participants receiving ***psychotherapy***. Leifer and Smith [91] presented quantitative data suggesting improvements in maternal psychosocial functioning, parent-infant interactions and secure attachment following therapy. Two qualitative case studies presented rich descriptions demonstrating the complexity of adult psychotherapy following severe trauma in infancy with thoughtful therapist reflections on the shared journey, including challenges for the therapist [41, 100].

### 5. Tools used during the perinatal period to identify parents with a history of childhood maltreatment and/or effects

We found 22 tools used to identify parents with a history of childhood maltreatment. These are briefly outlined below and summarised in Table 5. A more detailed assessment of psychometric values, the populations that tools have been validated within (including fathers), and barriers and facilitators for using tools will be addressed in a subsequent comprehensive review (S1 Appendix). The tools are categorised as those which; (a) exclusively assess an experience of traumatic event history (i.e. *exposure*), (b) assess experience of traumatic event history as part of a broader psychosocial needs assessment, (c) assess the *impact* of experience of traumatic event history as symptoms, (d) assess the *impact* of experience of traumatic event history on parenting, or (e) ‘other’.

**Table 5:** Tools used during the perinatal period for identifying parents with a history of child maltreatment and/or assessing effects: Evidence map.

Assessment tool (number of studies tool identified in)	Main domains measured	Characteristic Included Studies Table 1 Study IDs
**a. Tools for recognising traumatic event exposure only**		
Childhood Trauma Questionnaire (n = 9)	Emotional, physical and sexual abuse, and emotional and physical neglect.	2, 13a&b,15b*, 29a, 41a, 46b, 47a/b/c/c/e/f/j/k, 48a, 56a,c
Assessing environments III-History of parents’ childhood parenting experiences (n = 2)	Harsh and abusive parenting scale; physical punishment scale.	10, 28a
Adverse Childhood Experiences questionnaire (n = 3)	Ten forms of abuse, neglect, and household dysfunction before the age of 18.	19 a&b, 35*, 46a*b&c
Childhood Experience of Care and Abuse (n = 1)	CSA, physical abuse, psychological abuse, antipathy and neglect.	36a*
Brief Physical and Sexual Abuse Questionnaire (n = 1)	Quantifies severity of maternal violent trauma history.	49a/b
Child Abuse and Trauma Scale (n = 1)	38 items about the frequency and extent of negative childhood experiences, including the general atmosphere of their home and the way they felt they were treated in childhood by caregivers.	30
Early Trauma Inventory Self-Report Form (n = 1)	General trauma, and physical, emotional and sexual abuse.	50a
Trauma History Table Interview (n = 1)	Type, frequency, duration, and perpetrator identity for any child maltreatment experienced prior to age 16 years.	47c
Life Events Checklist (n = 1)	A 17-item checklist covering a range of potentially traumatic events from natural disasters to accidents, sudden losses to combat and interpersonal violent events.	49a&b
Life Stressor Checklist (n = 1)	Physical abuse, molestation, completed rape, emotional abuse, and physical neglect occurring prior to age 16.	47c
Childhood History Questionnaire (n = 1)	Presence and frequency of physical abuse, as well as witnessing abuse.	16
Antecedent Experiences Questionnaire (n = 1)	Childhood violence, neglect, or abuse experiences with severity ratings.	48#
Trauma section of American Indian Service Utilization and Psychiatric Epidemiology Risk and Protective Factors (AI-SUPERPFP) (n = 1)	Asks about 16 traumas; how often and at what age(s). Childhood (as opposed to adolescent) physical and sexual abuse were defined as experiences that occurred *prior to 13 years* of age.	38
**b. Tools for recognising traumatic event exposure as part of a broader psychosocial assessment**		
Brisbane Evaluation of Needs Questionnaire (n = 1)	History of childhood abuse of either parent is one of a range of risk factors.	5
Index of Need Questionnaire (n = 1)	Asks parents to identify if “you or your partner were physically and/or sexually abused as a child”.	23a, b&c
**c. Tools for assessing trauma symptoms**		
PTSD section of SCID (n = 3)	Exposure to involving actual or threatened death, serious injury, a threat to one’s physical integrity, or witnessing somebody else be killed or seriously harmed.	12, 15c, 49a&b
Post-traumatic Stress Diagnostic Scale (n = 1)	Traumatic event exposure and trauma symptoms, with many items corresponding to the PTSD section of the SCID.	18*
Adult attachment Interview (n = 6)	Assesses childhood relationships with attachment figures, usually parents.	26, 34#, 35*, 40a, 41a*, 46a*,
**d. Tools for assessing trauma symptoms among parents**		
Trauma Meaning Making Interview (n = 1)	Assesses post-traumatic change and explores maltreatment experiences and feelings, with questions added about the perceived impact of their child maltreatment experiences *on becoming a parent and the parent–child relationship*.	47c
Parent child conflicts-tactic scale/conflict-tactic scale (PC-CTS) (n = 3)_	Types of discipline *parents* received as a child, and if they ever experienced 11 parental disciplinary or neglectful actions.	11a&b, 53
Conceptual Change Questionnaire (CCQ) (n = 1)	Assesses how *parents* conceptualise abuse	54
**e. Other**		
Child Abuse Potential Inventory (n = 1)	160-item questionnaire designed to assist in the assessment of physical child abuse reports.	17

*primary validation data

^#^modifications

#### a) Tools which exclusively assess experience of traumatic event history (ie *exposure*)

The ***Childhood Trauma Questionnaire (CTQ)*** was reported in nine studies (20 articles) and measures emotional, physical and sexual abuse, and emotional and physical neglect domains [14, 20, 37, 51–53, 96, 105, 108–110, 113, 117, 118, 133, 135, 144, 145]. Blalock et al. [51] cites use of the tool in psychiatric, community, substance using, adolescent and adult populations. Test-retest reliability from birth to five years postpartum was also reported [55].

The ***Assessing Environments III-History of Parents’ Childhood Parenting Experiences*** tool [146] was used in two studies [46, 77]. Baydar et al. [46] described using a ‘harsh parenting scale’ (7 items) and an ‘abusive parenting scale’ (10 items), while Fornberg [77] reported using a ‘physical punishment scale’.

The ***Adverse Childhood Experiences (ACE)*** study questionnaire [147] was used in three studies (5 articles) in this review [61, 87, 104–106]. It retrospectively assesses exposure to ten forms of abuse, neglect, and household dysfunction before the age of 18. It was adapted and used in conjunction with the AAI in one study [104–106], with correlations reported between ACEs and the unresolved trauma category of the AAI [87, 104].

The ***Childhood Experience of Care and Abuse*** (CECA) [148] was used in one study [88] for comparing measures on the *Parental Bonding Instrument (PBI*) and includes assessments of CSA, physical abuse, psychological abuse, antipathy and neglect. The ***Brief Physical and Sexual Abuse Questionnaire*** (BPSAQ) [149] was used in one study [122, 150] to quantify the severity of maternal violent trauma history. The ***Child Abuse and Trauma Scale*** (CATS) [151] was used in one study [82] and consists of 38 items about the frequency and extent of negative childhood experiences, including the general atmosphere of their home and the way they felt they were treated in childhood by caregivers. The ***Early Trauma Inventory Self Report Short Form*** (ETI-SF) [151] was used in one study [123] and includes assessment of general trauma, and physical, emotional and sexual abuse.

The ***Trauma History Table Interview*** (THTI) was developed for the Maternal Anxiety in Childbearing Years (MACY) project [108–110] and assesses information about the type, frequency, duration, and perpetrator identity for any childhood maltreatment experienced prior to age 16 years [14]. The ***Life Events Checklist*** [152] as used in one study [122, 150] and is a 17-item checklist covering a range of potentially traumatic events from natural disasters to accidents, sudden losses to combat and interpersonal violent events. The ***Life Stressors Checklist*** [153] was used in one study [110] to assess trauma history. It is designed for use with women, and includes five items about childhood maltreatment (physical abuse, molestation, completed rape, emotional abuse, and physical neglect occurring prior to age 16). ***The Childhood History Questionnaire*** (CHQ) [154] was used by Bysom [57] to assess presence and frequency of physical abuse, as well as witnessing abuse. The ***Antecedent Experiences Questionnaire*** [155] was used by one study [94] with an adapted version of the AAI.

The ***Trauma section of the American Indian Service Utilization and Psychiatric Epidemiology Risk and Protective Factors Project*** (AI-SUPERPFP) interview [156] was used in a study with an American Native Indian community [92] and asks about 16 traumas; how often they occurred and at what age(s).

#### (b) Tools which assess experiences of traumatic event history as part of a broader psychosocial needs assessment

The ***Index of Need questionnaire*** developed and used in one study [68–70], asks parents to identify if “you or your partner were physically and/or sexually abused as a child”. The ***Brisbane Evaluation of Needs Questionnaire*** was used in another study [40] and includes childhood maltreatment screening questions.

#### (c) Tools which assess the symptoms of trauma

The ***Post-traumatic Stress Disorder section of the Structured Clinical Interview for DSM-IV* (SCID)** was used in conjunction with other tools in three studies (four articles) [9, 56, 122, 150] and assesses exposure to and effects of events involving actual or threatened death, serious injury, a threat to one’s physical integrity, or witnessing somebody else be killed or seriously harmed. Juul et al. [56] cites studies using the tool with mental health patients [157], college students and primary care patients [158].

The ***Posttraumatic Stress Diagnostic Scale (PDS)*** *[159]* was used in one study reporting screening burden and utility of the tool [59]. The PDS measures both trauma exposure and trauma-related symptoms, with many items corresponding to the PTSD section of the SCID.

The ***Adult Attachment Interview*** (AAI) [160] was identified in six studies [75, 86, 87, 94, 96, 97, 104]. The AAI assesses childhood relationships with attachment figures, usually parents. Attention is also paid to experiences with loss, abuse or other trauma, and a lack of resolution of trauma. Madigan et al. [96] assessed the stability of AAI attachment representations at three perinatal time points (prenatally, at infant age 6 to 9 months and at infant age 12 to 15 months) and found relatively low internal consistency for reporting the same experiences of loss, but higher consistency for reporting experiences of trauma.

#### (d) Tools that assess the *impact* of childhood maltreatment history on parenting

The ***Trauma Meaning Making Interview*** (TMMI) [161] was used in one study [14] to assess post-traumatic change and explore maltreatment in childhood experiences and feelings, with questions added about the perceived impact of their child maltreatment experiences. The ***Parent-Child Conflict Tactics Scale (PC-CTS)*** [162] was used in three studies [49, 50, 58] and includes questions about the types of discipline parent’s received as a child, and if they ever experienced any of 11 parental disciplinary or neglectful actions. The ***Conceptual Change Questionnaire*** (CCQ) (1999) was used in one study [128] to assess how parents conceptualise abuse.

#### e. Other tools

The ***Child Abuse Potential (CAP) inventory*** [163] was used in one study [58] and is a 160-item questionnaire designed to assist in the assessment of physical child abuse reports.

## Discussion

We located 57 studies conducted with participants during the perinatal period involving more than 20,000 parents. Most studies were conducted in the US and involved mothers only. No studies exclusively involved fathers, other family members or same-sex/gender diverse partners, and few included Indigenous parents. More than 75% of studies were categorised as ‘descriptive observational’ studies and we found no perinatal interventions specifically designed for parents with a history of childhood maltreatment and/or complex trauma.

Theories identified in our review are similar to those outlined in a clinical review for practitioners by [164]. However, our review included additional constructs that incorporated socioecological models to help explain some of the interactions and differences in parental outcomes. Studies in this review found post-traumatic changes were perceived by parents as predominantly positive during the perinatal period [14], supporting previous studies that suggest the parenting transition [165] offers a window of opportunity to transform the ‘vicious cycle of trauma’ into a ‘virtuous cycle of healing’ [166]. Most parents with a history of childhood maltreatment are able to provide nurturing environments for their children [44] and examining these ‘cycles of discontinuity’ is a promising place of exploration to illuminate innovative strategies for supporting parents [4]. Strategies parents reported using to support healing included; understanding and ‘meaning making’, conscious strategies to keep children safe and ‘parent differently’, increasing social support, spirituality and helping others. There were mixed reports about the value of psychotherapy and some parents expressed a desire for better access to more alternative therapies. A focus on these positive aspects and careful use of strengths-based language may also help to counteract some of the concerns about stigma, lack of trust and shame reported by parents in this review.

Studies in this review have reinforced National Trauma Guideline recommendations [6] by emphasizing the importance of considering childhood maltreatment and the sequelae of complex trauma within a socioecological context. This seems particularly important for Indigenous peoples with a histories of colonisation and ongoing discrimination. Our review also highlighted a number of system-level (e.g. lack of services, lack of transport, limited opening hours, use of waiting lists and long waiting times in clinics) and individual-level (e.g. fear of child protection agencies) contextual barriers.

Our findings support those of previous reviews that have identified the significance of the perinatal period in transmission of intergenerational trauma regarding the effects of stress [167], and a wide range of protective factors which are outside the mother-child relationship [168]. Studies in our review also have similarities with other reviews of intergenerational transmission of self-regulatory capacities and development of executive functioning skills in the first year of life [169, 170]. While much of the focus of childhood maltreatment is on the impact of experiences within the family, some studies suggest there are particular stages of development where children are more vulnerable to trauma from people outside the immediate family [171]. There is also a need to understand the impact of cumulative trauma as children start to explore and seek acceptance outside the family, in the broader community and society, and as they become parents; and the intersections between parental experiences of childhood maltreatment or complex trauma and societal racism that compound trauma [172].

No perinatal *intervention studies* were found specifically designed for parents with a history of childhood maltreatment. However, our findings are consistent with reviews not specific to the perinatal period or parents with a history of childhood maltreatment, that suggest parenting interventions can improve parent-infant interactions and attachment security [173–175]. *Intervention studies* of nurse home-visiting, parenting programs, IPT-B and CBT all suggested positive effects on parent wellbeing. However, more comprehensive meta-analysis of outcomes is needed in a full systematic review to articulate effect size estimates and explore sources of heterogeneity.

Parents with a history of childhood maltreatment often face specific social and emotional challenges during the perinatal period, and these understandings of social and emotional wellbeing are strongly shaped by culture. Thus it is essential that tools for measuring and assessing wellbeing are developed and validated among populations they are intended to be used by. There is a dearth of appropriate tools to measure concepts of social and emotional wellbeing among Indigenous people [176]. Often, Indigenous understandings of health and wellbeing strongly emphasize the collective and relational, incorporating dimensions of connectedness to family, community, culture, country and spirituality; alongside physical and mental wellbeing [177]. We found only one measurement tool and observational study involving Indigenous people [177]. We also found no studies involving only fathers [178] or extended family members. There is also a need to consider validity, reliability across the perinatal period, and utility [179]. There are multiple domains to measure, but practical considerations include feasibility [180], cost, time to administer, scoring ease, and sensitivity of questions [181]. We should also consider acceptability [181], parents’ perceptions [182], preferences [182], barriers and facilitators [183, 184].

To our knowledge, this is the first review to map evidence specific to the perinatal period involving parents with a history of childhood maltreatment. We used systematic methods including double screening all full-text articles, double data extraction and assessed risk of bias to inform the development of comprehensive protocols for in-depth reviews in the second phase of this work (S1 Appendix). The results of this scoping review have enabled development of a targeted search strategy for future reviews. It has mapped all index terms of included studies, searched similar studies on PubMed and developed word trees from included abstracts and titles. This scoping review has provided a summary ‘evidence map’ across all study types which is not characteristic of targeted reviews of single study designs.

Importantly, while full systematic reviews will take several years, this scoping review has provided important timely information that we have incorporated into a co-design process for developing perinatal strategies to identify and support Aboriginal Australian parents with a history of childhood maltreatment and/or complex trauma. For example, we have summarised protective and risk factors from the epidemiological studies, and themes from parent experiences and strategies on cards. Following interactive discussion groups with Elders and parents as part of an Intervention Mapping [185] framework, the card summaries were shared with an explanation that these issues emerged from studies with parents in other population groups, and asked parents to identify issues which may be relevant for them. In addition, we extracted and synthesized constructs from the 22 measurement tools which informed an interactive workshop activity with key stakeholders. In the workshop setting, we discussed the degree of importance of each of the main constructs, as well as issues associated with asking parents who have experienced childhood maltreatment about them during the perinatal period. Another major strength of this review is the inclusion of Aboriginal people in all stages of the review process to ensure culturally-relevant insight, in alignment with the United Nations Declaration on the Rights of Indigenous Peoples (UNDRIP) and Ethical conduct in research with Aboriginal and Torres Strait Islander Peoples and communities [186], and importantly balancing Indigenous worldviews with Western science.

There are limitations to this scoping review. First, our search may have missed intervention studies evaluating support strategies for parents that are embedded in general parenting programs. Second, only one reviewer conducted the preliminary title and abstract screen, which increases the likelihood of error. Third, we could not include detailed syntheses of findings specific to each study design, for example, thematic synthesis of qualitative studies and pooled analyses of epidemiological and intervention studies with sensitivity analysis to identify sources of heterogeneity or sub-group analyses to examine differential effects for subpopulations. We aim to address these limitations when we undertake in-depth systematic reviews (S1 Appendix).

There is little evidence that has demonstrated application of existing perinatal interventions for parents with a history of childhood maltreatment. Perinatal care services need to be ‘trauma-informed’ (aware) to minimise the risks of ‘triggering’ trauma responses for parents with a history of childhood maltreatment. The perinatal period offers a unique life-course ‘window of opportunity’ to recognise and assess parents who have experienced childhood maltreatment (and/or complex trauma) and enable access to trauma-specific support during frequent scheduled contacts with health services. For many predominantly healthy young people, starting a family may be the first contact with health services since their own childhood. It is also an important period for relational development, when many parents are motivated to make positive changes for the benefit of their child, and developing a nurturing relationship with the infant can generate positive responses that reinforce healing and ‘earned security’. Health experts have identified childhood maltreatment as a major public health challenge in mental health [187], with similar effects to those of tobacco on physical health [188]. In addition to significant associations with risk behaviours, it is likely that the effects of childhood maltreatment in the proposed criteria for complex PTSD (i.e. affective dysregulation, negative self-concept and interpersonal disturbances) [5], will have a differential and important impact public health program effectiveness. This includes how programs are received, engaged with and the capacity or agency that individuals have to respond to public health advice. There is an urgent need for all public health professionals to consider the impact of child maltreatment on health and health inequities more broadly [189].

## Conclusions

Growing observational and qualitative evidence and theories are available to inform development of trauma-informed perinatal services, recognition, assessment and trauma-specific support for parents who have experienced childhood maltreatment and/or complex trauma. There is continuing interest in developing theoretical constructs to explain phenomena, growing evidence of protective and risk factors and rich descriptions of parents experiences and strategies they use. However, evidence involving fathers, extended families, same-sex or gender diverse partners and Indigenous people is lacking. There has been little activity in applying existing evidence to support parents in the perinatal period. Incorporating socioecological and cultural contexts seems essential to informing effective perinatal strategies for recognising and supporting parents during this critical window of opportunity. Given the paucity of applied evidence, it is critical that strategies are developed in collaboration with families and communities, particularly for Indigenous peoples and fathers. Program design and evaluation strategies should incorporate short reflective cycles and flexible evaluation approaches to enable early detection of unforeseen circumstances and tailoring to respond to needs and reduce the risks of harm for vulnerable parents.

## Supporting information

S1 Appendix Perinatal awareness, recognition, assessment and support for parents who have experienced maltreatment in their own childhoods: Overview of reviews. (DOCX)

S2 Appendix PRISMA 2009 Checklist: Parenting after a history of childhood maltreatment: A scoping review and map of evidence in the perinatal period. (DOC)

S3 Appendix Healing the past by nurturing the future: Early support for parents who have experienced complex childhood trauma—A scoping review protocol. (DOCX)

S4 Appendix PsychInfo search strategy. (DOCX)

S5 Appendix Risk of bias assessment criteria. (DOCX)

S6 Appendix Assessment of study confidence using modified GRADE criteria. (DOCX)

S7 Appendix Table of excluded studies and reasons for exclusion. (DOCX)
