# Supplementary material for: Parenting after a history of childhood maltreatment: A scoping review and map of evidence in the perinatal period
Source: PLoS One. 2019 Mar 13;14(3):e0213460. doi: 10.1371/journal.pone.0213460 (PMC6415835; doi:10.1371/journal.pone.0213460)
Supplement: S1 Appendix — (DOCX) [file pone.0213460.s001.docx]

**S1 Appendix: Perinatal awareness, recognition, assessment and support for parents who have experienced maltreatment in their own childhoods: overview of reviews**

|  | **Phase 1: Mapping** | **Phase 2: In depth reviews** | | | | **Phase 3: Overview** |
| --- | --- | --- | --- | --- | --- | --- |
| **Population** | Parents planning pregnancy, during pregnancy or first two years postpartum | | | | | |
| **Primary review question** | What evidence is available regarding child maltreatment/complex childhood trauma during the perinatal period? | 1. What are the intergenerational pathways from parental maltreatment in the perinatal period? What factors mediate/moderate these outcomes? What theories help to explain these pathways (mechanisms)? And what aspects are supported or contradicted by the epidemiological evidence? | 1. What are perinatal experiences for parents who have experienced maltreatment in their own childhood? What strategies do parents use to heal and/or discontinue cycles of complex trauma? | 1. What is the effectiveness and cost of perinatal interventions for parents who have experienced maltreatment in their own childhood? Are there any differential effects of interventions in different subpopulations? | 1. What is the sensitivity, specificity and utility of assessment/screening tools used in the perinatal period for identifying parents who have experienced maltreatment in their own childhood (exposure) and/or trauma symptoms (effects)? | What works? For whom? In what circumstances? Are the most effective interventions also acceptable? What are the costs? |
| **Review type** | Scoping review | Systematic Review (epidemiological) | Systematic Review (qualitative) | Systematic review (quantitative) | Diagnostic/test accuracy review | Realist review |
| **Search** | ‘parent’ AND ‘childhood trauma’ AND ‘intergenerational’ AND ‘prevention’ | ‘parent’ AND ‘childhood trauma’ AND ‘intergenerational’ (based on revised terms from mapping phase) | | | | In-depth reviews, excluded reviews from previous search, integration with co-design workshops |
| **Study type** | Any primary study related to (theories; mediators/moderators; parents’ experiences; interventions; assessment/screening tools | Theoretical and epidemiological studies (observational). | Qualitative studies. | RCTs, CCTs, ITS (Descriptive studies). | Diagnostic/assessment/screening test accuracy studies. | Systematic reviews, co-design discussions |
| **Data extraction** | Microsoft Excel | Eppi-reviewer or Nvivo | | | | |
| **Synthesis** | Narrative synthesis | Narrative synthesis using socioecological model and integration with co-design workshop/qual studies with Elders. | Meta-synthesis of parents’ experiences (1^st^ level) and author conclusions (2^nd^ level) to generate unique review themes across studies (3^rd^ level). | Meta-analysis, meta-regression and narrative synthesis. Sensitivity analysis for major intervention components, study quality, implementation/process measures , and PROGRESS + characteristics (Age; Place; Race; education; social capital (partner/other); mental illness; SES; other risk factors). | HSROC analysis | Narrative synthesis |
| **Outcomes** | Evidence map | Diagram/illustration of resilience, protective and risk factors that mediate or moderate relationship between childhood trauma and behavioural & health outcomes for parents and infants. | Review level synthesis with GRADE-CERQual assessment of confidence in evidence. | Impact of interventions on process (acceptability/cost/implementation); parental behavioural and health outcomes; and infant behavioural and health outcomes. | Sensitivity and specificity of existing tools. | Recommendations for perinatal awareness, recognition, assessment and support strategies are likely to support resilience and healing for parents. |
