# Supplementary material for: Parenting after a history of childhood maltreatment: A scoping review and map of evidence in the perinatal period
Source: PLoS One. 2019 Mar 13;14(3):e0213460. doi: 10.1371/journal.pone.0213460 (PMC6415835; doi:10.1371/journal.pone.0213460)
Supplement: S3 Appendix — (DOCX) [file pone.0213460.s003.docx]

Healing the past by nurturing the future: Early support for parents who have experienced complex childhood trauma - a scoping review protocol

Chamberlain C, Gee G, Brennan S, Campbell S, Harfield S, Clarke Y, Glover K, Mensah F, Gartland D, Arabena K, Nicholson J, Brown S.

# Background

## Description of issue

Childhood abuse and neglect (maltreatment) is a global health priority affecting 25 to 50% of all children under 18 years of age worldwide (World Health Organisation, 2016). The World Health organisation (WHO) defines child maltreatment as “*all types of physical and/or emotional ill-treatment, sexual abuse, neglect, negligence and commercial or other exploitation, which results in actual or potential harm to the child’s health, survival, development or dignity in the context of a relationship of responsibility, trust or power*” (World Health Organisation, 2016). Childhood *trauma* includes child abuse and neglect, witnessing or experiencing domestic violence in childhood and other ‘adverse childhood experiences’ (Felitti et al., 1998; Kezelman and Stavropoulos, 2012). There is clear evidence that experiences of childhood trauma appears can have serious effects on the developing brain and impair the development of the nervous and immune systems’ leading to long term behavioural, physical and mental health problems in adulthood (World Health Organisation, 2016; Streeck-Fischer and van der Kolk, 2000; McCrory et al., 2010).

Trauma is often distinguished between ‘Type 1’ (acute exposure to a single event, with supportive adults to help child make sense of experience) and ‘Type 2’ or ‘complex trauma’. The latter refers to cumulative exposure to multiple traumatic experiences (Bath, 2008) that often involves interpersonal violation and occurs within the child’s care giving system. Children experiencing complex trauma are less likely to be supported in building resilience and recovery, and there is a higher risk for long term impacts on social and emotional wellbeing and development (Bath, 2008). Complex trauma (and the multiple negative impacts of child maltreatment) is not well described in the current fifth edition of the Diagnostic and Statistical Manual of Mental Disorders (DSM-V: American Psychiatric Association, 2013), and the diagnosis of Post-Traumatic Stress Disorder (PTSD) does not capture many dimensions of complex trauma (Kezelman and Stavropoulos, 2012; Alexander, 2016). Research often rely on reports of ‘child protection substantiations’ as a proxy measure for trauma, which is problematic for a number of reasons (Alexander, 2016)p7.

Experiences of complex childhood trauma and disrupted relationships with primary carer’s can impact on the capacity to develop social, emotional and cognitive skills necessary to form healthy interpersonal relationships throughout life, including with peers, partners and children (Streeck-Fischer and van der Kolk, 2000; Foley et al., 2000; Alexander, 2016). These effects are often framed using attachment theory to understand the impact of the disruption of the relationship between a child and primary carer (Alexander, 2016). However, other significant family and community relationships and broader societal factors also amplify or counteract the effects of trauma. The importance of context is evident in the differential rates of childhood trauma in disadvantaged populations, with strong associations between child maltreatment and poverty (Australian Institute of Health and Welfare, 2016). Hence, it is important that understandings of complex trauma are contextualised within children’s whole relational networks and social environments if it is to be adequately addressed (Kezelman and Stavropoulos, 2012). The WHO use an ecological framework (Dahlberg and Krug, 2002), to explain why some people are at higher risk of experiencing interpersonal violence, while others are more protected from it. This framework views interpersonal violence as the outcome of interactions among many factors at four levels—the individual, the relationship, the community, and the societal (figure 1) (Violence Prevention Alliance, 2016).


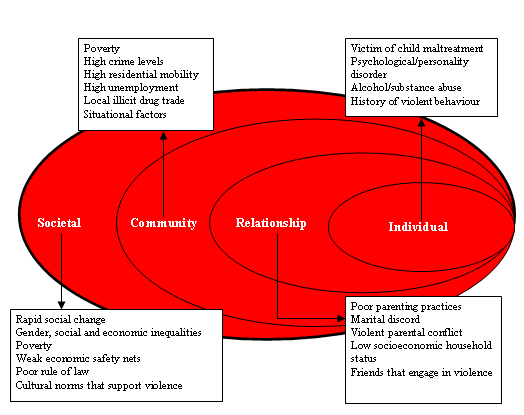


**Figure 1: The ecological framework and examples of risk factors at every level**

For example, in Australia, the rates of substantiated child maltreatment among Indigenous children are almost seven times higher than non-Indigenous children (Australian Institute of Health and Welfare, 2016). The reasons for the over-representation of Indigenous Australian children in child protection substantiations are complex and influenced by a legacy of past policies of forced removal, intergenerational effects of previous separations from family and culture, poverty and perceptions arising from cultural differences in child-rearing practices (Australian Institute of Health and Welfare, 2016). Atkinson (2002) describes how historical experiences of conflict and structural violence, child removals and discriminatory government interventions have resulted in increasing intra-family violence and psychological distress among Indigenous Australians, leading to ‘transgenerational’ or ‘intergenerational’ where trauma is transmitted from one generation to the next (Atkinson et al., 2010). Intergenerational transmission may be reflected in direct maltreatment, or the failure to protect the child from another perpetrator (Alexander, 2016)p11. Alexander (2016), using a family systems theory approach, argues for the importance of understanding trauma within families from an intergenerational perspective that acknowledges the impact and potential healing that can occur across generations. While many children who have experienced trauma survive and thrive, a study in New South Wales, Australia, found most children who have been exposed to childhood abuse and neglect (Australian Institute of Family Studies et al., 2015) experienced socio-emotional difficulties, particularly 12-17 year olds (Australian Institute of Family Studies et al., 2015).

### Parenting after complex childhood trauma

Transgenerational or intergenerational trauma theories recognise that the unresolved effects of trauma continue to impact adults when they become parents, with the primary carers’ moods, feelings, emotional reactions, and behaviours all having a big impact on the immediate and long-term wellbeing of children (Atkinson et al., 2010). Thus, unresolved intergenerational trauma is an important factor characterising difficult mother-child relationships (Furber et al., 2013). Additionally, parents, including pregnant women, who are traumatised may use alcohol or other drugs to help deal with trauma (Phillips, 2003), and the effects of these substances on the developing brain are well documented (McCrory et al., 2010). Amos and colleagues (2011) have used an attachment theory framework to describe individual-level aspects of the parent-child trauma related interactions and how a parent’s experiences can dramatically affect their parenting behaviour. In a secure care-giving relationship, a mother responds sensitively to her infants cues and needs for food, security and comfort. Amos (2011) describes how, in times of distress, an infant’s attachment system is activated and they rely on support from their caregiver to regulate these responses. If the parent withdraws or the response is confusing or hostile, conflicting attachment and defense systems are activated, leading to internal confusion and adaptive behavioural and relational problems (Amos et al., 2011), such as structural dissociation and experiential avoidance. These maladaptive responses can be maintained into adulthood as part of the trauma experience (Amos et al., 2011). Emotional fear responses can then be triggered when they become parent’s themselves, as they enter an attachment relationship with their own child and they respond to their child’s distress. Due to structural dissociation and avoidance, these responses are often re-experienced as conflicting sensations and emotions, rather than have a thought-out narrative (Amos etal 2011). This is turn can give rise to hostile or helpless responses to the growing child’s needs, as the parent simultaneously tries to manage distress associated with relational trauma, and the child’s attachment needs (Amos et al., 2015). For example, the inability to soothe a crying child can bring back memories of a parents experience of neglect (Alexander, 2016). If the parent is unable to overcome these responses, the child then experiences the same challenges as the parent and trauma is passed onto the next generation.

## Implications for health/rationale

Although parents who have experienced childhood trauma are at an increased risk of perpetuating intergenerational cycles of trauma compared to parents who have not experienced childhood trauma (Thornberry et al.), many parents who experience trauma are able to utilise natural resilience to overcome challenges (Alexander, 2015) and develop the necessary social and emotional skills to provide nurturing environments and care for their children. For example, in one study Bartlett and Easterbrooks (2015) found that 77% of maltreated mothers did not maltreat their own children (Dym Bartlett and Easterbrooks, 2015), with social support identified as a major protective factor. This reflects one of the most important findings from the growing research into the ‘neurobiology of attachment’, namely that healing can take place after severe experiences of childhood trauma, by restoring a sense of safety and well-being through nurturing, supportive relationships with others, and general enjoyment of life – a transition referred to as `earned security’ (Kezelman and Stavropoulos, 2012). Such findings have led to increasing calls from researchers in the area of child maltreatment to focus more on investigating processes related to the ‘discontinuities in the trauma cycle’ (i.e. protective factors and other moderators or ‘resilience’) (Alexander, 2016) which may disrupt harmful or endangering interactions within the parent-child relationship.

While pregnancy can be a joyful time, becoming a parent is also a major developmental challenge and vulnerable time, which can be stressful, especially where there are additional socioeconomic pressures and/or lack of social support (Bywood et al., 2015). Some subpopulations, such as people with low socioeconomic status and indigenous peoples, have higher risks of emotional distress and mental health conditions in the perinatal period, and these can impact on both the mother and child’s health (Bywood et al., 2015). These risks are compounded if there is unresolved childhood trauma as previously described, and the risk of intergenerational transmission increases. However, some evidence suggests that intergenerational transmission can be reduced with appropriate support to help the parent achieve a coherent perspective on childhood experiences and develop positive and supportive relationships (Kezelman and Stavropoulos, 2012; Alexander, 2016).

As well as reducing the intergenerational effects of trauma for children, interventions can support healing for the parent through development of positive, nurturing and loving relationships, and reduce the long term impacts. We suggest these ‘primary prevention’ screening and preventive interventions need to occur as early as possible before the child has already experienced trauma and the family are in crisis, and there can be considerable guilt and shame. Early intervention can enable a positive focus during an optimistic period, where the ‘vicious cycle’ of intergenerational trauma is disrupted and can become a ‘virtuous cycle’ that contains positively reinforcing elements (Segal et al., 2011). Segal and Dalziel present a schema which outlines the points of childhood trauma and consequences, and we highlight where intergenerational harms may be interrupted (Figure 2)(Segal and Dalziel, 2011).


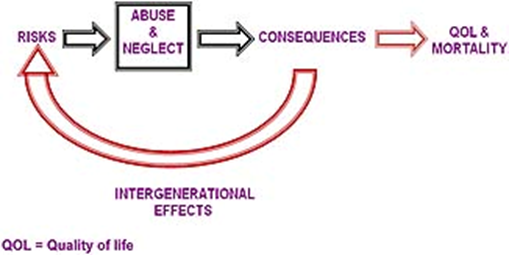


Interventions to prevent/disrupt the intergenerational effects for children, and mediate the negative effects for parents through development of nurturing loving relationships, leading to better long-term outcomes

**Figure 2: Risks and consequences of child abuse and neglect: a schema (Segal and Dalziel, 2011)**.

## Description of interventions and what has been done

In recognising the complexity and breadth of impact of childhood trauma, a recent ‘Lancet Commission’ on early childhood development promotes ‘nurturing care’ (caregiving, stimulation, responsiveness and safety) to assist children in reaching their developmental potential (Britto et al., 2016). Recommended strategies include a ‘multi-generational nurturing care package’, which ‘emphasises care and protection of the mother’s and father’s physical and mental health and wellbeing, while enhancing their capacity to provide nurturing care to their child.’ Generally, programs to support new or expectant parents aim to promote relationship skills and support, and realistic expectations of parenthood (Pinquart and Teubert, 2010). Interventions often focus explicitly on increasing sensitivity to the child’s needs and supporting parents to help the child feel safe. There are a number of reviews of interventions to support parents identified as having difficulties which have been developed and evaluated as highly cost effective (Pezzullo et al., 2010), including ‘Positive Parenting Program’, ‘Communities for Children’ and ‘Reconnect’ (Bywood et al., 2015; Bowes and Grace, 2014).

Guidelines have been developed which outline the principles of trauma-informed care in the general setting (Kezelman and Stavropoulos, 2012), but these are not specifically designed to provide support for parents who have experienced complex childhood trauma during the developmental transition to parenting. The United States National Centre for Trauma Informed Care (NCTIC) defines the core components of trauma-informed care as: understanding trauma and its impact; promoting safety; ensuring cultural competence; healing happens in relationships; integrating care; and understanding recovery is possible (Steele and Kuban, 2013). ‘Trauma-specific’ services are therapeutic responses to directly treat any symptoms or problems associated with trauma experiences (Kezelman and Stavropoulos, 2012). The core elements of complex trauma therapy generally involve understanding relevant neurobiological processes, engaging the right-brain, working with memories, attending to physical health and ‘phased treatment’ (Kezelman and Stavropoulos, 2012). As a child’s behaviour can sometimes trigger anxieties for parents who have unresolved trauma, and trauma-specific interventions aim to support parents through this process (Alexander, 2016). Therapy in relation to parenting also often includes ‘meaning making’ to understand and increase awareness of subconscious behaviours, context and effects; and to make conscious choices in parenting their own children (Alexander, 2016)p8.

Programs have been culturally adapted for new or expectant parents, including programs for Indigenous peoples that incorporate important cultural components such as the involvement of elders, traditional knowledge and holistic principles of social and emotional wellbeing (Closing the Gap Clearinghouse, 2013). While some programs designed to prevent intergenerational cycles of trauma exist, including among Indigenous communities (Victorian Aboriginal Health Service, 2014), there are few published evaluations of programs.

A current challenge is to integrate emerging evidence about the importance of trauma-informed health services and trauma-specific interventions and therapy (Kezelman and Stavropoulos, 2012), with evidence about interventions designed to support parents overcome the impacts of complex childhood trauma – and then to implement this in existing family planning/preconception, pregnancy and early childhood services.

## How interventions might work?

For many predominantly healthy young people of childbearing age, pregnancy may be the first point of contact with the health care system since childhood. Settings can include family planning/preconception clinics, general practitioners, maternity care, and early childhood services. This offers a unique ‘window of opportunity’ to ensure care systems for prospective and new parents are sensitive and responsive to experiences of childhood trauma (Kezelman and Stavropoulos, 2012). This includes support through the parenting journey which fosters resilience, enables healing and prevents the intergenerational transmission of trauma. Early preventive support is likely to be more constructive and positive when offered before any judgemental labels and negative associations of childhood neglect are involved. Importantly, for many who have experienced childhood trauma, pregnancy and impending parenthood may also act as an emotional trigger and it is important that health care professionals are able to identify people at risk and offer appropriate support.

In general, most programs to prevent intergenerational trauma are centred on supporting parents to build positive relationships and understand the effects of their own experiences. Programs often emphasize the critical role of all family members, particularly partners (Alexander, 2016). Recent research has found that the quality of a parent’s current relationship is instrumental in moderating the effects of intergenerational trauma. In particular, longitudinal studies (Schofield et al., 2013; Thornberry et al.) investigating parenting and the intergenerational transmission of child maltreatment have found that ‘stable nurturing relationships’ between both parents and children, and parents and other adults, can provide a protective, moderating effect. Some researchers have suggested that a focus on couple relationships may be more effective than a focus on parenting skills (Alexander, 2016). However despite this evidence, recent reviews suggest there are few interventions which focus on supporting fathers (Alexander, 2016)p79. (Alexander, 2015) has identified multiple sources of ‘natural resilience’ through examining discontinuities of intergenerational cycles of trauma, and suggests these can also be enhanced to reduce intergenerational effects. For example, a study that focussed on strategies employed by parents who have experienced childhood trauma themselves (Aparicio, 2016), found that enhancing social support and reducing isolation helped. The importance of social support has also been consistently noted in observational studies (Dym Bartlett and Easterbrooks, 2015; Dixon et al., 2009).

## Why it is important to do this review?

Complex childhood trauma can have profound long-term impacts on the capacity of parents to provide ‘nurturing care’ for their infants (Britto et al., 2016), which is critical to children reaching their full potential. Appropriate early prevention and intervention for prospective and new parents can help parents to resolve these challenges and prevent intergenerational transmission of trauma (van der Kolk, 2005).

While there is extensive literature on supporting families during childhood, including families with high risks (Bowes and Grace, 2014), there is limited literature outlining the evidence for identifying and providing early preventive support for people who have experienced complex childhood trauma before and during pregnancy and early childhood. Experts have concluded that interventions can be effective in interrupting intergenerational cycles of trauma, but recommend that these interventions should start early, preferably before the birth of the child (Alexander, 2016)p112.

One meta-analysis that included 143 studies of parenting education programs during pregnancy and the first 6 months after birth (Pinquart and Teubert, 2010) found that these interventions had “significant effects on parenting, child abuse/neglect, parental stress, health promoting parental behaviour and child development, parental psychological health, and couple adjustment.” However the review by Pinquart did not include screening prospective parents for complex childhood trauma, nor have a focus on specific strategies parents or service providers use to support parents to resolve trauma, and this scoping review will address this gap. A review of programs for children experiencing trauma found that trauma-focused cognitive behavioural therapy was ‘well supported’ by evidence (Gimson and Trewhella, 2014). (Bethell et al.) 2016 examined protective factors for childhood emotional, mental and behavioural problems after ‘adverse childhood experiences’ and recommended “integrated, family-centered, and mindfulness-based trauma-informed approaches” to address social and emotional trauma and interrupt intergenerational cycles of adverse childhood experiences and their impact on emotional, mental and behavioural problems among children and youth. However their recommendations did not focus on early prevention programs before or during pregnancy and early childhood. It is important to consider that while there is a strong rationale for screening, early identification and support, there are also potential risks associated with labelling individuals with ‘potential problems’ and undermining resilience and individual coping mechanisms inherent in many families.

This review will address these gaps with a focus on

- potential for screening around the time of pregnancy and early parenthood (up to 2 years postpartum) for previous childhood trauma
- early prevention and support strategies (around the time of pregnancy and early childhood up to 2 years) used by parents, or service providers to support parents who have experienced childhood trauma
- the acceptability and effectiveness (risks and benefits) for vulnerable subpopulations (e.g. Indigenous people.)

## Preliminary project scoping and consultation

This review aims to inform a broader program of work to develop effective strategies to support families to ‘break the cycle’ of intergenerational trauma. As such this review protocol is being developed in collaboration with, and co-designed by, clinical and community experts to ensure the questions are relevant and grounded in the reality of people’s experiences. Authors on this review are involved in various Indigenous family healing programs, including the developers of a program for Victorian Aboriginal parents who have experienced past trauma (Victorian Aboriginal Health Service, 2014) and the Indigenous Family Healing program (Bouverie Centre, 2016).

# Objectives

The aim is to scope existing evidence for identifying new (less than two years) or expectant parents that have experienced childhood trauma and providing support during pregnancy and early childhood to improve parental psychological wellbeing, child outcomes and prevention of subsequent childhood trauma.

The primary research question is:

1. Can interventions to identify and support parents who have experienced childhood trauma improve parental psychological wellbeing (e.g., healing), child wellbeing and prevent subsequent childhood trauma (e.g., disrupt intergenerational cycles)?

Secondary research questions include:

1. What tools have been used and validated to identify parents with previous childhood trauma? And in which populations?
2. Are there any differential effects of interventions in different subpopulations?
3. What strategies do parents use to heal and prevent transmission of intergenerational trauma for the child (i.e., ‘discontinuation’)?
4. What theories might help to inform development of strategies to support parents to heal and prevent transmission of intergenerational trauma for the child.

# Methods

## Criteria for inclusion

### Types of participants

Prospective and new (up to two years after birth) parents (mothers and fathers) or families where one of both parents, have experienced complex childhood trauma, including child abuse and neglect. Any measure of identification by investigators will be included.

Studies will be included where the age of the child is not specified or unclear, but participants are referred to as ‘parents’. However we will leave data extraction of these studies until last to check if they add any additional value. Studies where participants are not referred as parents will be excluded.

Studies will be included where any parents within the study have children younger than 2 years of age. When mean ages only are reported, studies reporting a mean age of less than five years only (ie preschool) will be included.

### Types of interventions

Any study which aims to:

1. identify parents who have experienced childhood trauma (screening (for childhood trauma experiences, impact and outcomes of prevention strategies))
2. describe factors which may help parents to heal and ‘discontinue’ cycles of trauma (e.g. mediators/moderators)
3. describe theories which may help develop strategies to support parents to heal and ‘discontinue’ cycles of trauma
4. and/or provide support before or during pregnancy (e.g. preconception and family planning clinics, medical services, maternity care services, and early childhood settings such as maternal-child health clinics and child-care centres) (up to two years of age).

Interventions will be subgrouped by the time the intervention *commences:*

- preconception, pregnancy up to 2 months postpartum, 2 to 12 months postpartum and 1 to 2 years postpartum.
- Main intervention strategies (e.g. counselling, social support, counselling and social support, other)

We acknowledge that there is likely to be considerable overlap between people experiencing current trauma/violence and those experiencing previous complex childhood trauma and we will collect and examine any reported information about current violence as a moderator of outcomes. However, studies which do not include an explicit aim to address previous childhood trauma with a focus on prevention will not be included in this review.

### Types of comparisons/studies

Any study design, including randomised controlled studies (RCTs), cluster RCTs, cohort studies (including screening studies), economic evaluations, qualitative studies. Reviews, guidelines, discussion and opinion papers, government reports and reports of primary studies which have not been peer-reviewed will not be included. However the reference lists of reviews will be checked to identify any additional studies.

### Types of outcomes

**Primary outcomes:**

- Measures of parental psychological wellbeing (trauma, anxiety and depression inventories, ‘healing’)
- Measures of childhood (< 5 years) wellbeing (developmental indicators (cognitive/speech), emotional/behavioural outcomes, trauma)
- Measures of parent-infant bonding/attachment and responsiveness

**Secondary outcomes:**

*Parental outcomes*

- Parental satisfaction and experiences of program
- Parental reports of independent strategies to overcome cycles of childhood trauma (discontinuation)
- Intimate partner violence reports
- Substance use
- Parenting self-efficacy and confidence
- Couple adjustment

*Child outcomes*

- Adverse childhood experiences (including abuse, trauma, neglect)
- Childhood hospitalisations (<2, 2-5,6+ yrs) including unintentional childhood injuries
- School entry educational measures
- Diagnosis of fetal alcohol syndrome
- Child protection referrals and substantiations
- Intentional childhood injuries

*Other outcomes*

- Theoretical evidence to explain impacts of complex trauma on parenting and/or therapies to promote recovery
- Economic evaluations
- Screening/identification tool validation measures (and comparisons of universal versus selective screening of ‘high risk’ populations)
- Adverse outcomes, such as harm or unintended consequences, disengagement with services, feelings of victimisation, labelling or undermining resilience
- Community violence

We will assess differential effects of interventions using PROGRESS equity framework: Place of residence; Race/ethnicity/culture/language; Occupation; Gender/sex; Religion; Education; Socioeconomic status; and Social capital. There is existing evidence that there are differential effects for mothers and fathers (Amos et al., 2015), so we will assess outcomes by gender where possible. We will also assess and other factors such as mental illness, young age (<20 years), current relationship violence (IPV), parental drug and alcohol use, and fetal alcohol syndrome disorder. However, we recognise that the inclusion criteria (childhood trauma) is strongly confounded by many of these factors. A logic model summarising the scoping review is outlined in Figure 3.

**Figure 3: Logic model for conceptualising scoping review analysis**

**Outcomes**

Parental, child and other outcomes

**Interventions**

Prevention, moderators, protectors, resilience, screening tools

**Population**

Prospective and new parents who have experienced complex childhood trauma

Differential effects by PROGRESS plus, including gender, ethnicity, mental illness, young age, current violence/trauma, drug and alcohol use, ‘at risk’ poula

## Search methods

We will search the following electronic databases for published literature using strategies that combine thesaurus terms and keywords relating to childhood trauma, prevention and parenting, with text word synonyms: Psycinfo, Medline, Cinahl and the Cochrane Library. We will use a search strategy developed and piloted in Psycinfo (see appendix 1) and subsequently modified for use in the remaining databases. The search included used a combination of synonyms for ‘child abuse’ AND ‘intergenerational’ AND ‘prevention’ AND ‘parent’. We will review the reference lists from included studies to look for new studies and assess index terms to determine if new words need to be added to the search strategy. We will also contact experts in the field.

References will be managed by using bibliographic reference management software (Endnote). Due to the high number of preliminary screening search results, one reviewer (CC) will independently screen titles and abstracts to identify potentially included documents. Text mining software will also be used and results screened by one reviewer to assess if any potentially included studies from the preliminary screen were missed.

The full texts of all potentially included studies will be retrieved and assessed by two reviewers according to the inclusion criteria below. Disagreements will be resolved by discussion or, if necessary, by a third reviewer. Studies which do not meet the inclusion criteria (eg population age or a review) will be coded as ‘relevant but excluded’.

## Data collection and analysis

### Data extraction and management

We will develop and pilot a data extraction tool in Microsoft Excel on two studies to systematically extract data on the:

- Study design (and quality) and setting
- Population characteristics (including PROGRESS+ domains and ‘at risk status)
- Intervention (including onset of intervention (subgrouped), intensity, delivery mode, staff qualifications, parental gender, risk status, main intervention strategy) and comparison characteristics
- Outcomes.

### Assessment of risk of bias

We will assess the risk of bias appropriate to the study design using adapted tools:

- RCTs and cluster RCTs – Cochrane risk of bias tool
- Controlled studies - Cochrane risk of bias tool with additional EPOC terms
- Cohort/observational studies (ROBINs)
- Qualitative (CASP tool)
- Assessment of screening tools (QUADAS checklist)
- Economic evaluations (Drummond checklist)

## Data synthesis

### Quantitative data

#### Measures of treatment effect/unit of analysis issues

In this scoping study, we anticipate there will be substantial heterogeneity in the types of study designs and thus will synthesize the outcomes narratively to provide an overall description of the body of relevant research evidence available.

We will synthesize outcomes narratively according to the outcome measures:

- - Measures to screen or identify parents who have experienced complex trauma
  - Factors which may moderate/mediate healing and/or transmission of intergenerational trauma
  - Theories about healing and/or transmission of intergenerational trauma
  - Descriptions of strategies to heal and/or interrupt the transmission of intergenerational trauma
  - Evaluations of strategies to heal and/or interrupt the transmission of intergenerational trauma
  - Descriptions of strategies parents themselves use to heal and/or interrupt intergenerational transmission of trauma
  - Any type of economic evaluation of strategies to heal and/or interrupt the transmission of intergenerational trauma
  - Other potentially relevant outcomes

We will tabulate the characteristics of included studies, and where possible, we will also tabulate a summary of the main outcomes for clarity.

#### Dealing with missing data

Where we have missing or unclear data or information, we will contact the investigators of the primary research.

# Acknowledgements

We greatly appreciate the advice and assistance with searching from Ms Poh Chua, librarian, Murdoch Research Children’s Institute.

NHMRC ECF grant no. 1088813.

# Contributions of authors

# Conflicts of interest

# References

National Collaborating Centre for Methods and Tools (2015). *PROGRESS Framework: Applying an equity lens to interventions*. Hamilton, ON: McMaster University. (Updated 20 July, 2015) Retrieved from <http://www.nccmt.ca/resources/search/234>.

Alexander P. (2015) *Intergenerational Cycles of Trauma and Violence: An Attachment and Family Systems Perspective* New York: W.W. Norton and Company.

Alexander P. (2016) *Intergenerational cycles of trauma and violence: An attachment and family systems perspective,* New York, NY: W.W. Norton & Company.

Amos J, Furber G and Segal L. (2011) Understanding maltreating mothers: a synthesis of relational trauma, attachment disorganization, structural dissociation of the personality, and experiential avoidance. *J Trauma Dissociation* 12: 495-509.

Amos J, Segal L and Cantor C. (2015) Entrapped Mother, Entrapped Child: Agonic Mode, Hierarchy and Appeasement in Intergenerational Abuse and Neglect. *Journal of Child and Family Studies* 24: 1442-1450.

Aparicio EM. (2016) ‘I want to be better than you:’ lived experiences of intergenerational child maltreatment prevention among teenage mothers in and beyond foster care. *Child & Family Social Work*: n/a-n/a.

Atkinson J, Nelson J and Atkinson C. (2010) Trauma, transgenerational transfer and effects on community wellbeing. In: Purdie N, Dudgeon P and Walker R (eds) *Working together: Aboriginal and Torres Strait Islander mental health and wellbeing practices and principles.* Canberra: Department of Health and Ageing.

Australian Institute of Family Studies, Chapin Hall Center for Children University of Chicago and New South Wales Department of Family and Community Services. (2015) Pathways of Care Longitudinal Study: Outcomes of children and young people in Out-of-Home care in NSW. Wave 1 baseline statistical report. Sydney: N.S.W: Department of Family and Community Services.

.

Australian Institute of Health and Welfare. (2016) Child protection Australia 2014–15. In: AIHW (ed). Canberra.

Bath H. (2008) The Three Pillars of Trauma Informed Care. *Reclaiming Children and Youth* 17: 17-21.

Bethell C, Gombojav N, Solloway M, et al. Adverse Childhood Experiences, Resilience and Mindfulness-Based Approaches. *Child and Adolescent Psychiatric Clinics* 25: 139-156.

Bouverie Centre. (2016) *Indigenous Family Healing Program*. Available at: <http://www.bouverie.org.au/the-indigenous-program/workin-with-the-mob-therapeutic-family-work-for-aboriginal-families>.

Bowes J and Grace R. (2014) Review of early childhood parenting, education and health intervention programs for Indigenous children and families in Australia. *Issues paper no. 8* Australian Institute of Family Studies for the Closing the Gap Clearinghouse.

Britto PR, Lye SJ, Proulx K, et al. (2016) Nurturing care: promoting early childhood development. *The Lancet*.

Bywood P, Raven M and Erny-Albrecht K. (2015) Improving health in Aboriginal and Torres Strait Islander mothers, babies and young children: A literature review. Adelaide: Primary Health Care Research & Information Service, Flinders University.

Closing the Gap Clearinghouse. (2013) Strategies and practices for promoting the social and emotional wellbeing of Aboriginal and Torres Strait Islander people. In: Clearinghouse PftCtG (ed) *Resource sheet no. 19.* Canberra & Melbourne: Australian Institute of Health and Welfare & Australian Institute of Family Studies.

Dahlberg L and Krug E. (2002) Violence-a global public health problem. In: Krug E, Dahlberg L, Mercy J, et al. (eds) *World Report on Violence and Health.* Geneva, Switzerland: World Health Organization, 1–56.

Dixon L, Browne K and Hamilton-Giachritsis C. (2009) Patterns of Risk and Protective Factors in the Intergenerational Cycle of Maltreatment. *Journal of Family Violence* 24: 111-122.

Dym Bartlett J and Easterbrooks MA. (2015) The moderating effect of relationships on intergenerational risk for infant neglect by young mothers. *Child Abuse Negl* 45: 21-34.

Felitti VJ, Anda RF, Nordenberg D, et al. (1998) Relationship of childhood abuse and household dysfunction to many of the leading causes of death in adults. The Adverse Childhood Experiences (ACE) Study. *Am J Prev Med* 14: 245-258.

Foley D, Goldfeld S, McLoughlin J, et al. (2000) A review of the early childhood literature. In: Health CfCC (ed). Canberra: Department of Family & Community Services.

Furber G, Amos J, Segal L, et al. (2013) Outcomes of Therapy in High Risk Mother-Child Dyads in Which There is Active Maltreatment and Severely Disturbed Child Behaviors. *Journal of Infant, Child, and Adolescent Psychotherapy* 12: 84-99.

Gimson K and Trewhella A. (2014) Developing a Trauma-Informed therapeutic service in the Australian Capital Territory for children and young people affected by abuse and neglect. Canberra: ACT Community Services Directorate, Office for Children Youth and Family Support, Early Intervention and Prevention Services.

Kezelman C and Stavropoulos P. (2012) Practice Guidelines for Treatment of Complex Trauma and Trauma Informed Care and Service Delivery. Sydney: Adults Surviving Child Abuse.

McCrory E, De Brito S and Viding E. (2010) Research review: The neurobiology and genetics of maltreatment and adversity. *The Journal of Child Psychology and Psychiatry* 51: 1079-1095.

Pezzullo L, Taylor P, Mitchell S, et al. (2010) Positive Family Functioning. Canberra: Access Economics Pty Limited.

Phillips G. (2003) Addictions and healing in Aboriginal country. Canberra: Aboriginal Institute of Aboriginal and Torres Strait Islander Studies.

Pinquart M and Teubert D. (2010) Effects of parenting education with expectant and new parents: a meta-analysis. *J Fam Psychol* 24: 316-327.

Schofield TJ, Lee RD and Merrick MT. (2013) Safe, Stable, Nurturing Relationships as a Moderator of Intergenerational Continuity of Child Maltreatment: A Meta-Analysis. *The Journal of adolescent health : official publication of the Society for Adolescent Medicine* 53: S32-S38.

Segal L and Dalziel K. (2011) Investing to Protect Our Children: Using Economics to Derive an Evidence-based Strategy. *Child Abuse Review* 20: 274-289.

Segal L, Doidge J and Amos J. (2011) Determining the determinants: Is child abuse and neglect the underlying cause of the socio-economic gradient in health? In: Callaghan L (ed) *Determining the Future: A Fair Go and Health for All.* Redland Bay, QLD: Connor Court.

Steele W and Kuban C. (2013) *Working with Grieving and Traumatised Children and Adolescents: Discovering what matters most through Evidence-Based Sensory Interventions,* New Jersey: John Wiley and Sons Inc.

Streeck-Fischer A and van der Kolk B. (2000) Down will come baby, cradle and all: Diagnostic and therapeutic implications of chronic trauma on child development. *Australian and New Zealand Journal of Psychiatry* 34: 903-918.

Thornberry TP, Henry KL, Smith CA, et al. Breaking the Cycle of Maltreatment: The Role of Safe, Stable, and Nurturing Relationships. *Journal of Adolescent Health* 53: S25-S31.

van der Kolk B. (2005) Developmental Trauma Disorder: Toward a rational diagnosis for children with complex trauma histories. *Psychiatric Annals* 35: 401-408.

Victorian Aboriginal Health Service. (2014) *Breaking the Cycle of Trauma, Koori Parenting, What Works for Us*. Available at: <http://www.vahs.org.au/koori-parenting-resources/> Accessed 16/9/2016.

Violence Prevention Alliance. (2016) *The ecological framework*. Available at: <http://www.who.int/violenceprevention/approach/ecology/en/>.

World Health Organisation. (2016) Child maltreatment factsheet. Geneva: WHO.

# Appendix 1: PsycInfo search strategy

| **Main concepts (combined vertically with AND)** | **Synonyms (combined with OR): subject heading and key word search** | **Comments/results** |
| --- | --- | --- |
| Childhood trauma | 1. exp child abuse/ or child neglect/ or exp child welfare/ or complex ptsd/ or emotional abuse/ or physical abuse/ or exp sexual abuse/ or verbal abuse/  2. (child* adj (abuse or neglect or maltreatment or trauma or welfare or trauma or (domestic adj violence) or advers* or (toxic adj stress) or (adverse adj2 experience*))).ti,ab,id. |  |
| Intergenerational cycles | 3. early experience/  4. transgenerational patterns/ or intergenerational relations/ or exp parent child relations/  5. (exposure or pattern*1 or cycle* or inter-generational or intergenerational or trans-generational or transgenerational or generation* or relational or transmission or (offspring adj victimi#ation) or continuity).ti,ab,id. |  |
| Prevention/protective factors | 6. (moderat* or protect* or prevent* or discontinuit* or buffer* or break* or resilien* or heal* or recovery).ti,ab,id.  7. "resilience (psychological)"/ or coping behavior/ or exp emotional adjustment/ or emotional stability/ or posttraumatic growth/ or protective factors/ or psychological endurance/  8. prevention/ or exp intervention/  9. adjustment/ or exp emotional adjustment/ or social adjustment/ or "adaptability (personality)"/ or adaptive behavior/ |  |
| Parent | 10. exp parents/ or exp expectant parents/  11. (mother* or parent* or father* or pregnan* or antenatal or antepartum* or postnatal or postpartum or prenatal).ti,ab,id. |  |

12. (1 or 2) and (3 or 4 or 5) and (6 or 7 or 8 or 9) and (10 or 11)

**Medline search strategy 3/11/2016**

1. exp Child Abuse/

2. (child* adj (abuse or neglect or maltreatment or trauma or welfare or (domestic adj violence) or advers* or (toxic adj stress))).tw,kf,hw.

3. Intergenerational Relations/

4. (exposure or pattern*1 or cycle* or inter-generational or intergenerational or trans-generational or transgenerational or generation* or relational or (early adj experience*) or transmission or (offspring adj victimi#ation) or continuity).tw,kf,hw.

5. pc.fs.

6. exp social support/

7. (moderat* or protect* or prevent* or discontinuit* or buffer* or break* or resilien* or heal* or recovery).tw,kf,hw.

8. exp social adjustment/ or exp social skills/

9. exp parent-child relations/

10. exp fathers/ or exp mothers/ or exp single parent/

11. (mother* or parent* or father or pregnan* or antenatal or antepartum* or postnatal or postpartum or prenatal or maternal or paternal).tw,kf,hw.

12. (1 or 2) and (3 or 4 or 9) and (5 or 6 or 7 or 8) and (10 or 11)

**Cinahl Ebsco host**

(((MH+%26quot%3bChild+Abuse%2b%26quot%3b))+OR+((child*+W1+(abuse+OR+neglect+OR+maltreatment+OR+trauma+OR+welfare+OR+(domestic+W1+violence)+OR+advers*+OR+(toxic+W1+stress)))))+AND+(((MH+%26quot%3bIntergenerational+Relations%26quot%3b))+OR+((exposure+OR+pattern*+OR+cycle*+OR+inter-generational+OR+intergenerational+OR+trans-generational+OR+transgenerational+OR+generation*+OR+relational+OR+transmission+OR+(offspring+W1+victimi%3fation)+OR+continuity))+OR+((MH+%26quot%3bParent-Child+Relations%2b%26quot%3b)))+AND+(((MH+%26quot%3bSocial+Adjustment%26quot%3b))+OR+((moderat*+OR+protect*+OR+prevent*+OR+discontinuit*+OR+buffer*+OR+break*+OR+resilien*+OR+heal*+OR+recovery))+OR+((MH+%26quot%3bEarly+Intervention%26quot%3b)+OR+(MH+%26quot%3bCrisis+Intervention%26quot%3b)+OR+(MH+%26quot%3bHardiness%26quot%3b)))+AND+(((MH+%26quot%3bParents%2b%26quot%3b))+OR+((mother*+OR+parent*+OR+father*+OR+pregnan*+OR+antenatal+OR+antepartum*+OR+postnatal+OR+postpartum+OR+prenatal+OR+maternal+OR+paternal)))

**Cochrane Library search strategy:**

ID Search

#1 MeSH descriptor: [Child Abuse] explode all trees

#2 child* next (abuse or neglect or maltreatment or trauma or welfare or "domestic violence" or advers* or "toxic stress") (Word variations have been searched)

#3 MeSH descriptor: [Intergenerational Relations] explode all trees

#4 MeSH descriptor: [Parent-Child Relations] explode all trees

#5 exposure or pattern*1 or cycle* or inter-generational or intergenerational or trans-generational or transgenerational or generation* or relational or "early experience*" or transmission or "offspring victimisation" or "offspring victimization" or continuit* (Word variations have been searched)

#6 MeSH descriptor: [Social Support] explode all trees

#7 MeSH descriptor: [Social Adjustment] explode all trees

#8 moderat* or protect* or prevent* or discontinuit* or buffer* or break* or resilien* or heal* or recovery (Word variations have been searched)

#9 MeSH descriptor: [Social Skills] explode all trees

#10 MeSH descriptor: [Emotional Adjustment] explode all trees

#11 MeSH descriptor: [Parents] explode all trees

#12 mother* or parent* or father or pregnan* or antenatal or antepartum* or postnatal or postpartum or prenatal or maternal or paternal (Word variations have been searched)

#13 (#1 or #2) and (#3 or #4 or #5) and (#6 or #7 or #8 or #9 or #10) and (#11 or #12)

**Pubmed search**

("child abuse" OR "child neglect" OR "child maltreatment" OR "child welfare" OR "domestic violence" OR "adverse childhood experiences" OR "toxic stress") AND ("intergenerational relations" OR exposure OR pattern* OR cycle* OR inter-generational OR intergenerational OR trans-generational OR transgenerational OR generation* OR relational OR early experience* OR transmission OR "offspring victimisation" OR "offspring victimization" OR continuit*) AND ("social support" OR "social adjustment" OR "social skills" OR moderat* OR protect* OR prevent* OR discontinuit* OR buffer* OR break* OR resilien* OR heal* OR recovery) AND (mother* OR parent* OR father OR pregnan* OR antenatal OR antepartum* OR postnatal OR postpartum OR prenatal OR maternal OR paternal) AND ((NOTNLM OR publisher[sb] OR inprocess[sb] OR pubmednotmedline[sb] OR indatareview[sb] OR pubstatusaheadofprint))
