# Supplementary material for: Parenting after a history of childhood maltreatment: A scoping review and map of evidence in the perinatal period
Source: PLoS One. 2019 Mar 13;14(3):e0213460. doi: 10.1371/journal.pone.0213460 (PMC6415835; doi:10.1371/journal.pone.0213460)
Supplement: S4 Appendix — (DOCX) [file pone.0213460.s004.docx]

**S4 Appendix: PsychInfo search strategy**

| **Main concepts (combined vertically with AND)** | **Synonyms (combined with OR): subject heading and key word search** |
| --- | --- |
| Childhood trauma | 1. exp child abuse/ or child neglect/ or exp child welfare/ or complex ptsd/ or emotional abuse/ or physical abuse/ or exp sexual abuse/ or verbal abuse/  2. (child* adj (abuse or neglect or maltreatment or trauma or welfare or trauma or (domestic adj violence) or advers* or (toxic adj stress) or (adverse adj2 experience*))).ti,ab,id. |
| Intergenerational cycles | 3. early experience/  4. transgenerational patterns/ or intergenerational relations/ or exp parent child relations/  5. (exposure or pattern*1 or cycle* or inter-generational or intergenerational or trans-generational or transgenerational or generation* or relational or transmission or (offspring adj victimi#ation) or continuity).ti,ab,id. |
| Prevention/protective factors | 6. (moderat* or protect* or prevent* or discontinuit* or buffer* or break* or resilien* or heal* or recovery).ti,ab,id.  7. "resilience (psychological)"/ or coping behavior/ or exp emotional adjustment/ or emotional stability/ or posttraumatic growth/ or protective factors/ or psychological endurance/  8. prevention/ or exp intervention/  9. adjustment/ or exp emotional adjustment/ or social adjustment/ or "adaptability (personality)"/ or adaptive behavior/ |
| Parent | 10. exp parents/ or exp expectant parents/  11. (mother* or parent* or father* or pregnan* or antenatal or antepartum* or postnatal or postpartum or prenatal).ti,ab,id. |

12. (1 or 2) and (3 or 4 or 5) and (6 or 7 or 8 or 9) and (10 or 11)
