# Supplementary material for: Parenting after a history of childhood maltreatment: A scoping review and map of evidence in the perinatal period
Source: PLoS One. 2019 Mar 13;14(3):e0213460. doi: 10.1371/journal.pone.0213460 (PMC6415835; doi:10.1371/journal.pone.0213460)
Supplement: S5 Appendix — (DOCX) [file pone.0213460.s005.docx]

**S5 Appendix: Risk of bias assessment criteria**

| Criteria (numbered by ROB tool source*) | RCT | CCT | ITS | Cohort | Qual | Screen | Other |
| --- | --- | --- | --- | --- | --- | --- | --- |
| 1. Was the allocation sequence adequately generated? (H/L/U) |  |  |  |  |  |  |  |
| 1. Was allocation adequately concealed? (H/L/U) |  |  |  |  |  |  |  |
| 1. Were there any baseline imbalances? (H/L/U) |  |  |  |  |  |  |  |
| 1. Were incomplete outcome data adequately addressed? (bias due to missing data) Were withdrawals explained and justified? *Assessments should be made for each main outcome (or class of outcomes).* (H/L/U) |  |  |  |  |  |  |  |
| 1. Was knowledge of the allocated intervention adequately prevented during the study (blinding)? Participants (H/L/U). Could knowledge of the intervention have led to providers or others altering the intervention in some way (H/L/U). Could knowledge of the intervention have led to those involved in outcome assessment altering the way outcomes were assessed (H/L/U) |  |  |  |  |  |  |  |
| 1. Was the study adequately protected against contamination? (eg exposure of control group) (H/L/U) |  |  |  |  |  |  |  |
| 1. Are results reported according to intervention allocation? (ITT) H/L/U |  |  |  |  |  |  |  |
| 1. Are reports of the study free of suggestion of selective outcome reporting? (H/L/U) |  |  |  |  |  |  |  |
| 1. ITS: Was the intervention independent of other changes? (H/L/U) |  |  |  |  |  |  |  |
| 1. ITS: Was the shape of the intervention effect pre-specified? (H/L/U) |  |  |  |  |  |  |  |
| 1. ITS: Was the intervention unlikely to affect data collection? (H/L/U) |  |  |  |  |  |  |  |
| 1. Are there any concerns about the outcome measures? (H/L/U) |  |  |  |  |  |  |  |
| 2. 2. Are confounders adequately identified and accounted/adjusted for? (H/L/U) |  |  |  |  |  |  |  |
| 2. 3. Bias in selection of participants into the study? (H/L/U) |  |  |  |  |  |  |  |
| 2 .3. Bias in classification of interventions |  |  |  |  |  |  |  |
| 2. 3. Bias due to deviations from intended interventions |  |  |  |  |  |  |  |
| 4. Qualitative methodology appropriate? (H/L/U) |  |  |  |  |  |  |  |
| 4. Recruitment/sampling strategy appropriate for aims? (H/L/U) |  |  |  |  |  |  |  |
| 4. Data collection addresses the research issue? (H/L/U) |  |  |  |  |  |  |  |
| 4. Relationship between researcher and participant considered? (H/L/U) |  |  |  |  |  |  |  |
| 4. Ethical issues considered? (H/L/U) |  |  |  |  |  |  |  |
| 5.Was the sample size adequate? (H/L/U) |  |  |  |  |  |  |  |
| 5. Does the data analysis seem appropriate? (H/L/U) |  |  |  |  |  |  |  |
| 5. Was the spectrum of patients representative of the patients who will receive the test in practice? (H/L/U) |  |  |  |  |  |  |  |
| 1. Were selection criteria clearly described? (H/L/U) |  |  |  |  |  |  |  |
| 1. Is the reference standard likely to correctly classify the target condition? (H/L/U) |  |  |  |  |  |  |  |
| 1. Is the time period between reference standard and index test short enough to be reasonably sure that the target condition did not change between the two tests? (H/L/U) |  |  |  |  |  |  |  |
| 1. Did the whole sample or a random selection of the sample, receive verification using a reference standard of diagnosis? (H/L/U) |  |  |  |  |  |  |  |
| 1. Did patients receive the same reference standard regardless of the index test result? (H/L/U) |  |  |  |  |  |  |  |
| 1. Was the reference standard independent of the index test (i.e. the index test did not form part of the reference standard)? (H/L/U) |  |  |  |  |  |  |  |
| 1. Was the execution of the index test described in sufficient detail to permit replication of the test? (ie all test results sensitivity/specificity reported) (H/L/U) |  |  |  |  |  |  |  |
| 1. Was the execution of the reference standard described in sufficient detail to permit its replication? (ie all test results sensitivity/specificity reported) (H/L/U) |  |  |  |  |  |  |  |
| 1. Were the index test results interpreted without knowledge of the results of the reference standard? (Blinded?) (H/L/U) |  |  |  |  |  |  |  |
| 1. Were the reference standard results interpreted without knowledge of the results of the index test? (blinded) (H/L/U) |  |  |  |  |  |  |  |
| 1. Were the same clinical data available when test results were interpreted as would be available when the test is used in practice? (H/L/U) |  |  |  |  |  |  |  |
| 1. Were uninterpretable/ intermediate test results reported? (H/L/U) |  |  |  |  |  |  |  |
| Could the inclusion of this study potentially bias the generalisability of the review? ROB: H/L/U Equity pointer: Remember to consider whether disadvantaged populations may have been excluded from the study. (H/L/U) |  |  |  |  |  |  |  |
| Other sources of bias (H/L/U) |  |  |  |  |  |  |  |

**Source/references:**

1. Higgins, J. P. T., Altman, D. G., Gøtzsche, P. C., Jüni, P., Moher, D., Oxman, A. D., . . . Sterne, J. A. C. (2011). The Cochrane Collaboration’s tool for assessing risk of bias in randomised trials. BMJ, 343.
2. Cochrane Effective Practice and Organisation of Care (EPOC). (2017). Suggested risk of bias criteria for EPOC reviews. Retrieved from <http://epoc.cochrane.org/resources/epoc-resources-review-authors>
3. Sterne, J. A., Hernán, M. A., Reeves, B. C., Savović, J., Berkman, N. D., Viswanathan, M., . . . Higgins, J. P. (2016). ROBINS-I: a tool for assessing risk of bias in non-randomised studies of interventions. *BMJ, 355*.
4. Critical Appraisal Skills Programme. (2017). CASP Qualitative checklist. Retrieved from <http://docs.wixstatic.com/ugd/dded87_25658615020e427da194a325e7773d42.pdf>
5. Whiting, P. F., Rutjes, A. W., Westwood, M. E., Mallett, S., Deeks, J. J., Reitsma, J. B., . . . Bossuyt, P. M. (2011). QUADAS-2: a revised tool for the quality assessment of diagnostic accuracy studies. Annals of Internal Medicine, 155(8), 529-536. doi:10.7326/0003-4819-155-8-201110180-00009
