# Supplementary material for: Parenting after a history of childhood maltreatment: A scoping review and map of evidence in the perinatal period
Source: PLoS One. 2019 Mar 13;14(3):e0213460. doi: 10.1371/journal.pone.0213460 (PMC6415835; doi:10.1371/journal.pone.0213460)
Supplement: S6 Appendix — (DOCX) [file pone.0213460.s006.docx]

**S6 Appendix: Assessment of study confidence using modified GRADE criteria**

|  | **Grade down one or two levels for each of the following domains for which there are serious (-1) or very serious (-2) concerns (final GRADE: High, moderate, low, very low)*.** |
| --- | --- |
| Intervention studies (RCT/CCTs etc) | 1. **Study limitations** (concerns about sequence generation; allocation concealment; blinding; intention to treat/incomplete outcome data adequately addressed)  2. **Indirectness** (concerns about outcome measure validation)  3. **Imprecision** (concerns about adequate sample size [using a rule of thumb: 400 or less events for binary outcomes or participants for continuous outcomes**] or data analysis) |
| Interrupted time series | NA (no studies) |
| Observational studies | 1. **Study limitations** (concerns about selection bias; incomplete outcome data adequately addressed; adequate accounting/adjustment for confounders)  2. **Indirectness** (concerns about outcome measure validation)  3. **Imprecision** (concerns about sample size or data analysis) |
| Assessment/diagnostic/screening test studies | 1. **Study limitations** (concerns about patient representativeness; complete outcome data; reference standard validation)  2. **Indirectness** (concerns about appropriateness or validity of outcome measures)  3. **Imprecision** (concerns about sample size or data analysis) |
| Qualitative Studies | 1. **Methodological limitations** (concerns about whether qualitative method is appropriate or researcher-participant relationship considered)  2. **Relevance** (concerns whether data collection addresses research issue)  3. **Adequacy of data** (concerns about sampling strategy; analysis approach or data richness) |

* based on modified GRADE approach. Inconsistency and publication bias not assessed because GRADE was applied to single studies. All studies start from ‘High’ in this scoping review, including observational studies (i.e. High epidemiological evidence).

** based on Murad, M. H., Mustafa, R. A., Schunemann, H. J., Sultan, S., & Santesso, N. (2017). Rating the certainty in evidence in the absence of a single estimate of effect. *Evidence Based Medicine, 22(3*), 85-87.
