# Supplementary material for: Parenting after a history of childhood maltreatment: A scoping review and map of evidence in the perinatal period
Source: PLoS One. 2019 Mar 13;14(3):e0213460. doi: 10.1371/journal.pone.0213460 (PMC6415835; doi:10.1371/journal.pone.0213460)
Supplement: S7 Appendix — (DOCX) [file pone.0213460.s007.docx]

**S7 Appendix: Table of excluded studies and reasons for exclusion**

| **Study ID** | **Reason for exclusion** |
| --- | --- |
| Adamakos 1986 | No parental childhood trauma |
| Akai 2008 | No parental childhood trauma |
| Alexander 2016 | Book chapter. |
| Alford 2015 | Does not involve parents in first 2 years postpartum. |
| Alink 2009 | No parental childhood trauma |
| Allbaugh 2014 | Mean age of children was 12 years old. |
| Allen 2008 | Describes impact of different types of psychological abuse on adult mental health conditions (not parents) |
| Alsop 1996 | College students not parents. |
| Amos 2011 | No parental childhood trauma. |
| Amos 2015 | No parental childhood trauma. |
| Amos 2015_Seep94 | Conference abstract only |
| Anderson 2006 | Theories of resilience of adult survivors, but not specifically parents. |
| Anderson 2011 | Adult women reporting what helped their post-traumatic growth and recovery from childhood trauma. |
| Appleyard 2003 | Includes recommendations for therapy for parents experiencing PTSD, not specifically complex childhood trauma |
| Ashburn 2016 | Not specifically for recovery from complex childhood trauma, but intervention to de-normalise family violence among men in Uganda. |
| Atkinson 2011_chapter10 | Book chapter. |
| Austin 2010_Dissertation | No parental childhood trauma. Analysis of revictimization among survivors of child abuse. |
| Bahm 2017 | No parental childhood trauma. |
| Bailey 2009 | Children aged 5+. Shows multiple predictors of continuity for harsh parenting across G1, G2 and G3. |
| Baker 2001 | Children aged 5-19. |
| Banaschewski 2011 | Editorial/commentary. |
| Baril 2016 | Children over 24mths. |
| Barlev 2005 | Participants are college students not parents. |
| Barlow 2007 | Protocol only for preventing recurrence of emotional abuse. |
| Barlow 2013 | Group parenting program review (not childhood trauma). |
| Barlow 2014-The_Cochrane_Library | No childhood trauma. Parenting intervention review. |
| Barlow 2015-The_Cochrane_Library | No parental childhood trauma. |
| Barnes 2013 | No parental childhood trauma. |
| Barnet 2012 | No parental childhood trauma. |
| Bavolek 2000 | Editorial. |
| Bavolek 2012 | Book summary. |
| Bavolek 2016 | Parenting intervention brochure |
| Begle 2011 | Parenting intervention for parents of children aged 3-6 years. |
| Beissel 2008 | Child aged 2-5 years. |
| Belsky 2005 | Doesnt discuss childhood trauma (only harsh and sensitive parenting). |
| Belsky 2009 | Editorial for special edition. |
| Belt 1996 | Not parents. |
| Belt 1998 | Children mean age 6 years. |
| BenDavid 2015 | Not parents. |
| Bergamo 2011 | Not in English. |
| Bernard 2002 | Systematic review. |
| Bernstein 1994 | Not parents. Validation paper of most used CT measure (CTQ). |
| Bert 2009 | Did not describe mediating or moderating factors. |
| Biaggi 2015 | Systematic review. |
| Bigfoot 2011/12 | No parental childhood trauma. Includes cultural adaptation of a child trauma project for AI communities, and also describes different ways of working appropriately with AI families (ie more active assistance and less talk). |
| Bishop 1999 | Participants are mothers who have perpetrated abuse not specifically victims of complex trauma. |
| Blizard 2006 | Editorial. |
| Bolton 2006 | Qualitative study exploring survivors relationships with their parents (participants not specifically parents themselves). |
| Borelli 2015 | Not parents. |
| Borrego 2008 | Not childhood trauma. |
| Bottos 2014 | Article withdrawn from publication. |
| Bouros 2012 | Not in English. |
| BowerRusser 2005 | Participants are college students. |
| Bradfield 2011 | Not parents. |
| Bradfield 2013 | Not parents. |
| Bramblett 1998 | Fathers of older children. |
| Brent 2013 | Systematic review. |
| Brewer 2004 | Award description. |
| Bridgett 2015 | Systematic review. |
| Briere 2001 | Participants children. |
| Brink 2001 | Book abstract. |
| Britton 1998 | Abstract only. |
| Buchanan 1998 | Systematic review. |
| Burton 2004 | Editorial. |
| Busch 2010 | Book abstract only. |
| Calheiros 2001 | Not in English. |
| Campbell 1996 | Mean age of children 9 years. |
| Candib 2012 | Discusses impact of trauma among doctors (not explicitly parents) |
| Carroll 2013 | Not specifically parents with children less than 2 years of age. |
| Casse 2015 | Describes self control/self-efficacy among mothers who were institutionalised as adolescents (not specifically childhood trauma) |
| Cloitre 2014 | Not parents. |
| Conger 2009 | Editorial |
| Connolly 2011 | Review of theories of intergenerational trauma transmission. |
| Cook 1995 | Psychological impact of childhood trauma among college students. |
| Cornille 2006 | Unclear if participants had experienced childhood trauma (Describes curriculum for DADs program). |
| Cox 1995 | Not parents. Reflection on resilience factors as viewed by survivors. |
| Cukor 2004 | Not parents. Describes childhood trauma and psychological factors among a sample of psychiatric patients. |
| Cukor 2006 | Not parents. Reports mediating effects of cognitive style on relationship between childhood trauma and poor psychiatric outcomes. |
| Danieli 2015 | Not childhood trauma. Explores impact of holocaust trauma on children and grandchildren of survivors. |
| DeGregio 2012 | Systematic eview. |
| Dehay 2009 | Adolescent children. |
| Devoe 2002 | Systematic review. |
| Dietz 1999 | Not parents. Reports association between child abuse and unintended pregnancy in adulthood. |
| Douglas 2000 | Children Aged just under 5 to 10 years. |
| Downey 1987 | Not complex trauma. |
| Downey 1997 | Book. |
| Downs 1992 | Not parents. |
| Downs 1998 | Not parents. Describes association betweeen child abuse and low self-esteem. |
| Draucker 1997 | Not parents in perinatal period. Describes mediating roles of cognition and social support between child abuse and victimization |
| Dube 2004 | Not parents in perinatal period. But screening for ACE and assesses screening tool accuracy. |
| Dubowitz 2011 | Not parents in perinatal period. |
| Dunivan 2013 | Not parents in perinatal period. Describes increased acceptance of deception among child abuse victims. |
| Dunn 1993 | Not parents in perinatal period. Describes experiences of 9 people growing up with psychotic mother. |
| Dunn2001 | Not parents in perinatal period. Proposed that paternal substance use and neglect would cause psychological dysregulation in child but this was not demonstrated. |
| Dutra2009 | Participants 19 years of age. |
| Edgeland 1988 | Book. |
| Ehrensaft2015 | Mean age 8.3 years. Discusses impact of CT on parenting. |
| Ehrensaft2016 | Mean age 8.3 years. IPV predicts more negative and less supportive parenting practices. |
| England2005 | Not parents in perinatal period. Describes issues around caring for cognitively impaired elderly parents after CT. |
| Eshed2008 | Not parents in perinatal period. Qualitative information on 7 themes from 3 married fathers who broke cycle of trauma. |
| Etter2013 | Not parents in perinatal period. Summary of impact of CT on adolescents. |
| Fantuzzo 2007 | No parental child maltreatment - study includes subsample of parents who have maltreated their own children already. |
| Felitti 1998 | Not parents in perinatal period. |
| Ferch 2001 | Abstract only. |
| Fergusson 1997 | Not parents in perinatal period. |
| Fisher 2006 | Not parents in perinatal period. Grand-daughters of incest victims 'co-research' impact of their mothers trauma on them. |
| Fonagy 2006 | Not parents in perinatal period. Discusses how violence develops from childhood experiences. |
| Font 2016 | Not parents in perinatal period. Child physical, emotional, and sexual abuse were significantly associated with several adult health risks, beyond the effects of other adversities. |
| Ford 2001 | Systematic review. |
| Fuller-Thomson 2014 | Population over 18 so likely to include parents, but not specified that they are. |
| Giordano 2011 | Not parents in perinatal period. Impact of working with child abuse survivors on therapists. |
| Giordano 2013 | Not parents in perinatal period. Impact of working with child abuse survivors on therapists. |
| Gold 1994 | Not parents in perinatal period. Women who were sexually assaulted in childhood scored higher on dissociation and sexual problems. |
| Green-Miller 2012 | 10 women recovering from addiction who have daughters (age unclear). Thesis investigates transformative power of parenting transition among women experiencing addiction. 10 women recovering from addiction who have daughters (age unclear). Thesis investigates transformative power of parenting transition among women experiencing addiction. |
| Greer 1994 | 10 women recovering from addiction who have daughters (age unclear). Thesis investigates transformative power of parenting transition among women experiencing addiction. Case study of recovery of adult survivor of child sexual abuse |
| Gunn 2006 | Not specifically childhood trauma. Describes 'ANEW' intervention to improve service providers confidence in discussing psychosocial issues during pregnancy |
| Hall 1998 | Age of children not stated. |
| Hall 2011 | Adult children. |
| Hanley 1996 | Not parents in perinatal period. Description of factors associated with poor parental attachment (aged 3-6 years) among adult survivors of child sexual abuse. |
| Hardt 2010 | Not parents in perinatal period. Describes validation of retrospective ACE screening tool using two large national datasets. |
| Hatzinikolaou 2016 | Not specifically childhood trauma. Describes evaluation of "Parent–Infant Relationship Global Assessment Scale" among parents assessed as 'high risk of abuse' and 'not high risk of abuse' aged about 1-3 years. |
| Hefler 1987 | Systematic review. Describes interventions to support parents with CT during the perinatal period, in context with available literature (in 1987). |
| Henscel 2014 | Children aged 2.3-3.7 years. |
| Herzog 1992 | Not parents in perinatal period. |
| Higgins 2000 | Not parents in perinatal period. Describes impact of multiple types of maltreatment in adulthood |
| Hollander-Goldfein 2012 | Book. |
| Hulette 2011 | Age of children 7-8 years. Historical summary of research on trauma recovery. |
| Huxtable_Jester 2005 | Not parents in perinatal period. Describes mediating role of adult attachment on intergenerational transmission of child abuse. |
| Issokson 2004 | Book chapter discussing impact of abuse on pregnancy and childbearing. |
| Jaffee 2013 | Not parents in perinatal period. Important findings about the role of safe, stable, nurturing relationships between intimate partners and between mothers and children are associated with breaking the cycle of abuse in families. |
| Jones 2008 | Book chapter. Describes father's experience of healing from abusive tendencies. |
| Keager 2010 | No explicit history of parental childhood trauma. Describes how motherhood is an important transitional period for criminal desistance. |
| Kerr 2009 | Not parents in perinatal period. Pathways of parenting patterns across 3 generations. |
| Kezelman 2015 | Trauma guideline. |
| Kilpatrick 2005 | Not parents in perinatal period. Validation of parental empathy measures among parents registered with child protection and controls |
| Kleiner 2002 | Not parents in perinatal period. Describes program to support teenagers with incarcerated parents, and advocates for stronger parent/family support on release from prison as a strategy for reducing re-offending |
| Ko 2008 | Systematic review of support for adolescents experiencing trauma. |
| Kovan 2009 | No information on mediators/moderators. Observational study of intergenerational transmission of parenting styles assessed at 24 months postpartum. |
| Kreager 2010 | No explicit history of parental childhood trauma. The transition to motherhood is the primary turning point for disadvantaged women to exit delinquent and drug using trajectories. |
| Krecklewetz 1998 | Not parents in perinatal period. Study exploring how child sexual abuse survivors parent their pre-adolescent children |
| Langeland 1995 | Systematic review describing factors associated with discontinuation of complex trauma. |
| Lieberman 2011 | Systematic review outlining implications of childhood trauma and early childhood interventions. |
| Litrownik 2013 | Editorial: concise overview of evidence of SSNRs in mediating intergenerational transmission of trauma. |
| MacFarland 2004 | Not parents in perinatal period. Thesis using creative methods to explore the role of nature in healing from trauma. |
| Main 1984 | Discusses 'harsh parenting practices' (not specifically trauma). |
| Maxwell 2016 | Not parents in perinatal period. Findings suggest that psychotherapeutic experiences after experiencing childhood physical abuse may decrease the likelihood of perpetrating violence in adulthood. |
| Mayer 2010 | Book section. Case study of therapy to reduce intergenerational transmission. |
| McNeill 1999 | Children aged 3+. |
| Merrick 2013 | Editorial introducing special edition on SSNRs. |
| Min 2013 | Describes association between maternal childhood trauma and child behaviour problems at 9 years of age. |
| Mullins Geiger 2014 | Policy review paper examining current programs and policies targeting pregnant and parenting youth “aging out” of the child protection system. |
| Murphy 2016 | Not parents in perinatal period. Proposes implementation of trauma-informed care in paediatrics. |
| Neckoway 2011 | Not specifically parents in perinatal period who have experienced trauma. Describes Canadian Ojibway perspectives of parenting and issues related to child protection placements. |
| Neppl 009 | Not parents in perinatal period. Describes parenting practice continuity across 2 generations and mediating factors among sample of adolescents. |
| Norman 2012 | Systematic review and meta-analysis of health problems after child maltreatment. |
| Ondersma 2006 | Validation of child abuse potential inventory in general US population (not parents). |
| Paolillo 2006 | Focussed on intimate partner relationships not childhood trauma. Describes experiences and perspectives of men exposed to DV as children who did not go on to perpetuate abusive intimate partner relationships. |
| Parker 1997 | Describes 'dysfunctional parenting' screening tool validation (not childhood trauma). |
| Plant 2013 | Studies impact on adolescents. Offspring of mothers who experienced both maternal childhood maltreatment and antenatal depression were exposed to significantly greater levels of childhood maltreatment and exhibited significantly higher levels of adolescent antisocial behaviour compared with offspring not so exposed. |
| Pulido 2001 | Editorial about importance of addressing DV during pregnancy. |
| Richter 2016 | Lancet report outlining importance of investment in nurturing care programs. |
| Riser 2009 | Not parents in perinatal period (children over 2 years of age). Explores associations between parent trauma, parenting style and child trauma. |
| Rivera 2015 | Not parents in perinatal period - emerging adults in romantic relationships. Explores role of maternal forgiveness in perpetuation of domestic violence. |
| Roussillon 1998 | Systematic review of considerations in perinatal care for child sexual abuse survivors. |
| Runtz 1997 | Examines impact of CT among psychological wellbeing of university students. |
| Russell 2016 | Describes strong association between CT and eating disorders. |
| Scaramella 2003 | No parental exposure to trauma, although 'harsh parenting' assessed. |
| Schecter 2003 | Book chapter. |
| Scholfield 2013 | Systematic review and meta-analysis of the role of SSNRs. |
| Shaffer 2009 | Not parents in perinatal period. Social competence mediated the intergenerational relation of parenting quality. personality (i.e., constraint). |
| Siegel 2013 | Systematic review arguing for a trauma-informed approach to identifying children and parents whose symptoms of emotional dysregulation may be otherwise overlooked. |
| Sotero 2006 | Systematic review of trauma theory. |
| Stewart-Brown 2005 | Describes adult risk factors following child abuse |
| Sugishita 2013 | No clear PCT - Survey of perinatal mental health support in Japan. |
| Tarabulsy 2016 | Editorial for special edition on Canadian contributions to Attachment theory. |
| Tarzcon 2004 | A literature review with summary of childhood trauma research. |
| Thompson 2010 | Book chapter. |
| Thornberry 2012 | Systematic review of intergenerational trauma research questioning cycle hypothesis. |
| Tilmans 1995 | Not in English. |
| Tracy 2006 | Book chapter. |
| Uslucan 2009 | Book chapter describing theories of attachment and intergenerational transmission of violence. |
| Verona 2005 | Age of children unclear but appear to be older than 2 years. |
| Wangarin 1994 & 1996 | Describes protective parenting tendencies among CSA survivors but no children aged 2 or less. |
| Wilen 2013 | Campbell Collaboration protocol for psychosocial interventions to support adults sexually abused as children. |
| Wingo 2010 | Not parents in perinatal period. Found resilience moderates depressive symptom severity in individuals exposed to childhood abuse or other traumas both as a main effect and an interaction with trauma exposure. |
| Zalewski 2013 | Psychiatrically ill children aged 7-16. Concludes that when treating psychiatrically ill children, it is important for a child’s clinician to consider mothers’ childhood abuse histories in addition to their history of depression. |
| Zeanah 2011 | Review of clinical implications of attachment research, including circle of security. |
